# Characteristics of Immediate-Early 2 (IE2) and UL84 Proteins in UL84-Independent Strains of Human Cytomegalovirus (HCMV)

**DOI:** 10.1128/Spectrum.00539-21

**Published:** 2021-09-22

**Authors:** Salome Manska, Cyprian C. Rossetto

**Affiliations:** a Department of Microbiology and Immunology, Reno School of Medicine, University of Nevada, Reno, Nevada, USA; Barnard College, Columbia University

**Keywords:** UL84, cytomegalovirus, herpesvirus, immediate-early 2 (IE2), viral DNA synthesis

## Abstract

Human cytomegalovirus (HCMV) immediate-early 2 (IE2) protein is the major transactivator for viral gene expression and is required for lytic replication. In addition to transcriptional activation, IE2 is known to mediate transcriptional repression of promoters, including the major immediate-early (MIE) promoter and a bidirectional promoter within the lytic origin of replication (*ori*Lyt). The activity of IE2 is modulated by another viral protein, UL84. UL84 is multifunctional and is proposed to act as the origin-binding protein (OBP) during lytic replication. UL84 specifically interacts with IE2 to relieve IE2-mediated repression at the MIE and *ori*Lyt promoters. Originally, UL84 was thought to be indispensable for viral replication, but recent work demonstrated that some strains of HCMV (TB40E and TR) can replicate independently of UL84. This peculiarity is due to a single amino acid change of IE2 (UL122 H388D). Here, we identified that a UL84-dependent (AD169) Δ84 viral mutant had distinct IE2 localization and was unable to synthesize DNA. We also demonstrated that a TB40E Δ84 IE2 D388H mutant containing the reversed IE2 amino acid switch adopted the phenotype of AD169 Δ84. Further functional experiments, including chromatin-immunoprecipitation sequencing (ChIP-seq), suggest distinct protein interactions and transactivation function at *ori*Lyt between strains. Together, these data further highlight the complexity of initiation of HCMV viral DNA replication.

**IMPORTANCE** Human cytomegalovirus (HCMV) is a significant cause of morbidity and mortality in immunocompromised individuals and is also the leading viral cause of congenital birth defects. After initial infection, HCMV establishes a lifelong latent infection with periodic reactivation and lytic replication. During lytic DNA synthesis, IE2 and UL84 have been regarded as essential factors required for initiation of viral DNA replication. However, previous reports identified that some isolates of HCMV can replicate in a UL84-independent manner due to a single amino acid change in IE2 (H388D). These UL84-independent strains are an important consideration, as they may have implications for HCMV disease and research. This has prompted renewed interest into the functional roles of IE2 and UL84. The work presented here focuses on the described functions of UL84 and ascertains if those required functions are fulfilled by IE2 in UL84-independent strains.

## INTRODUCTION

Human cytomegalovirus (HCMV), a betaherpesvirus, has a large double-stranded (ds) DNA genome of approximately 246,000 bp, with over 200 putative open reading frames. HCMV is found worldwide, with most people infected during their lifetime through direct contact with infected bodily fluids. In the United States, age-adjusted seroprevalence is 58.9%, increasing from 36.3% in 6- to 11-year-olds to 90.8% in individuals over 80 years old (1). HCMV infection is primarily asymptomatic in healthy hosts. However, in immunocompromised or immune immature individuals, HCMV can cause severe disease (2–4). Uncontrolled lytic replication in immunocompromised hosts can lead to HCMV-related pneumonia, retinitis, colitis, and in some cases death (5). HCMV is also the leading congenital infection in the United States, resulting in a wide range of disorders, including hearing loss, vision loss, microcephaly, intellectual disabilities, developmental delays, and other neurologic abnormalities (6–8).

A hallmark for all herpesviruses is the ability to establish a latent infection after primary infection and remain with the infected individual throughout the life of the host. During latency, there are periodic episodes of reactivation followed by lytic replication and release of new virions. During lytic replication, HCMV encodes a set of six core proteins required for DNA synthesis. These proteins are: UL54 (DNA polymerase), UL44 (polymerase processivity factor), UL105 (helicase), UL70 (primase), UL102 (primase-associated factor), and UL57 (single-stranded DNA-binding protein). These core proteins were identified using the transient-transfection replication assay (9, 10). The transient-transfection replication assay is a method in which a plasmid containing the *cis*-acting *ori*Lyt is cotransfected with plasmids that encode viral factors required for origin-dependent DNA replication. The *cis*-acting HCMV *ori*Lyt is a complex replicator composed of a mix of G+C repeats, transcription factor binding sites, a bidirectional promoter, and a region containing an RNA stem-loop structure (11–15). In conjunction with the six core proteins, the cotransfection replication assay revealed that HCMV required a total of 11 viral factors to replicate *ori*Lyt, two of those factors being immediate-early 2 (IE2) and UL84 (10). The other viral factors (UL36-38, UL112-113, and IRS1/TRS1) proved to be dispensable in other cell types by replacing the native promoters with constitutive promoters or using core replication machinery from other herpesviruses. However, UL84 and IE2 were factors seemingly required for *ori*Lyt-dependent replication, presumably by acting as initiator proteins or origin-binding proteins (OBP) (16, 17).

The major viral transactivator during lytic replication is the IE2 86-kDa (IE2-86) protein. IE2-86 is transcribed from the major immediate-early (MIE, UL122-123) locus. In addition to IE2-86, the MIE locus encodes additional proteins that are expressed by either alternative splicing or alternative ATG start sites; these include IE1-72, IE2-40, and IE2-60. IE2-86 has been extensively studied, and its importance to the progression of lytic replication is highlighted by its ability to regulate viral gene expression and also participate in viral DNA replication. Many studies of IE2 have focused on its interaction with UL84. Additionally, IE2 is known to govern UL84 expression (18).

Previous characterizations of IE2 and UL84 were identified using bacterial artificial chromosome (BAC)-derived mutant viruses with deletions of either the IE2 or UL84 locus. For example, Towne BAC deletions within IE2 (MIE exon 5, UL122) failed to replicate due to lack of lytic gene expression (19, 20). Similar results were obtained from an AD169 BAC with a small mutation within exon 5 of IE2 (21). Mutants of UL84 in AD169 and Towne strains were also unable to produce virus or plaques when BAC DNA was transfected into permissive primary human fibroblasts (20, 22). Additionally, an AD169 BACmid containing a disruption to the nuclear export signal (NES) of UL84 rendered UL84 unable to shuttle between the nucleus and cytoplasm and was also unable to replicate (23, 24).

While UL84 is required for strains AD169, Towne, FIX, Merlin, and Toledo, it was discovered that a single amino acid mutation in IE2 can substitute for the dependence of UL84 in HCMV strains TB40E and TR (25). The ability of TB40E to replicate without UL84 due to a mutation within IE2 requires us to reevaluate the roles and functions that had previously been ascribed to UL84 and IE2. Understanding how these proteins contribute to infection will further elucidate the mechanism of initiation during lytic HCMV DNA synthesis. Therefore, in this work we investigated the roles of IE2 and UL84 relating to *ori*Lyt-dependent DNA replication and transactivation of early viral promoters.

## RESULTS

### Phenotype of UL84 deletion in UL84-dependent and UL84-independent strains.

**FIG 1:**
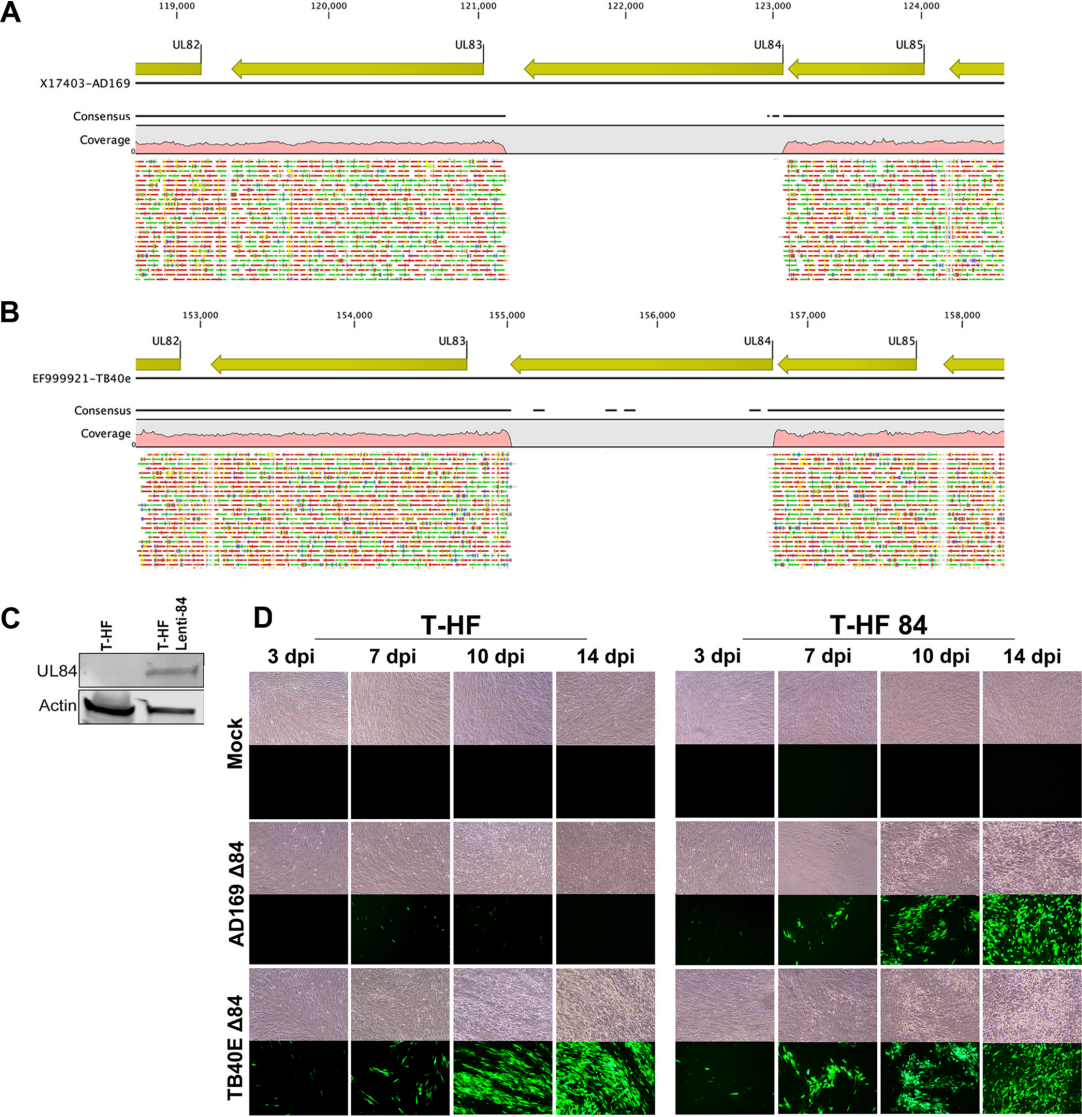
Genotype and phenotype analysis of AD169 Δ84 and TB40E Δ84. AD169 Δ84 and TB40E Δ84 mutants were generated using BAC recombineering. Whole-genome sequencing was performed to confirm deletion of the UL84 locus. Qiagen CLC genomics workbench was used to map reads from mutant sequences to the HCMV reference genome. (A) Mapping of AD169 Δ84 to the reference genome showed deletion in UL84. (B) Mapping of TB40E Δ84 to the reference genome showed deletion in UL84. (C) A T-HF 84 cell line was generated by transducing T-HF cells with lentivirus encoding UL84 and selected for puromycin resistance. Total cell lysate was harvested and protein was resolved by SDS-PAGE. Protein was detected using antibodies specific to UL84 and actin as a cellular load control. (D) HF and T-HF 84 cells were infected with either TB40E Δ84 or AD169 Δ84 (MOI = 1). Cells were imaged using light and fluorescence microscopy at 3 dpi, 7 dpi, 10 dpi, and 14 dpi as indicated. Images were taken at ×10 magnification.

To characterize the role of UL84 and IE2, experiments were performed in both UL84-dependent (AD169) and UL84-independent (TB40E) HCMV strains. Initial experiments included generating mutants that contained a deletion of the UL84 locus in both AD169 and TB40E by BAC recombineering. Whole-genome sequencing analysis of the AD169 Δ84 and TB40E Δ84 mutants verified successful deletion of UL84 (Fig. 1A and B). To determine the phenotypic attributes of these mutant viruses, primary human fibroblast (HF) cells and a cell line stably expressing the UL84 protein (T-HF 84) were used for infection studies. To generate the complementing cell line expressing UL84, life-expanded telomerase human fibroblast cells (T-HF) were transduced with a lentivirus containing the UL84 gene expressed from the eF1α promoter. Following transduction, cells were selected using puromycin. Production of UL84 in the T-HF 84 cells was detected by Western blotting (Fig. 1C). The T-HF 84 cell line was used to complement UL84-dependent mutants and generate infectious virus for downstream assays.

Since the AD169 Δ84 and TB40E Δ84 mutants were generated using HCMV BACmids containing a green fluorescent protein (GFP) expression cassette, this allowed for viral infection to be detected by the presence of GFP expression. Additionally, viral infection results in cytopathic effects (CPE) in fibroblasts, which are observable under light microscopy. The T-HF cells and T-HF 84 cells were infected (MOI = 1) with AD169 Δ84 or TB40E Δ84 (Fig. 1D). By 7 days postinfection (dpi), both AD169 Δ84 and TB40E Δ84 in T-HF and T-HF 84 cells show GFP-positive cells, indicating the establishment of infection. But unlike TB40E Δ84, which shows spread on T-HF and T-HF 84 cells at 10 and 14 days, AD169 Δ84 was able to spread only on T-HF 84 cells and not on the T-HF cells. By using T-HF 84 cells to compensate for a lack of genomic UL84, these data support the previous reports that AD169 requires UL84 for virus replication.

**FIG 2:**
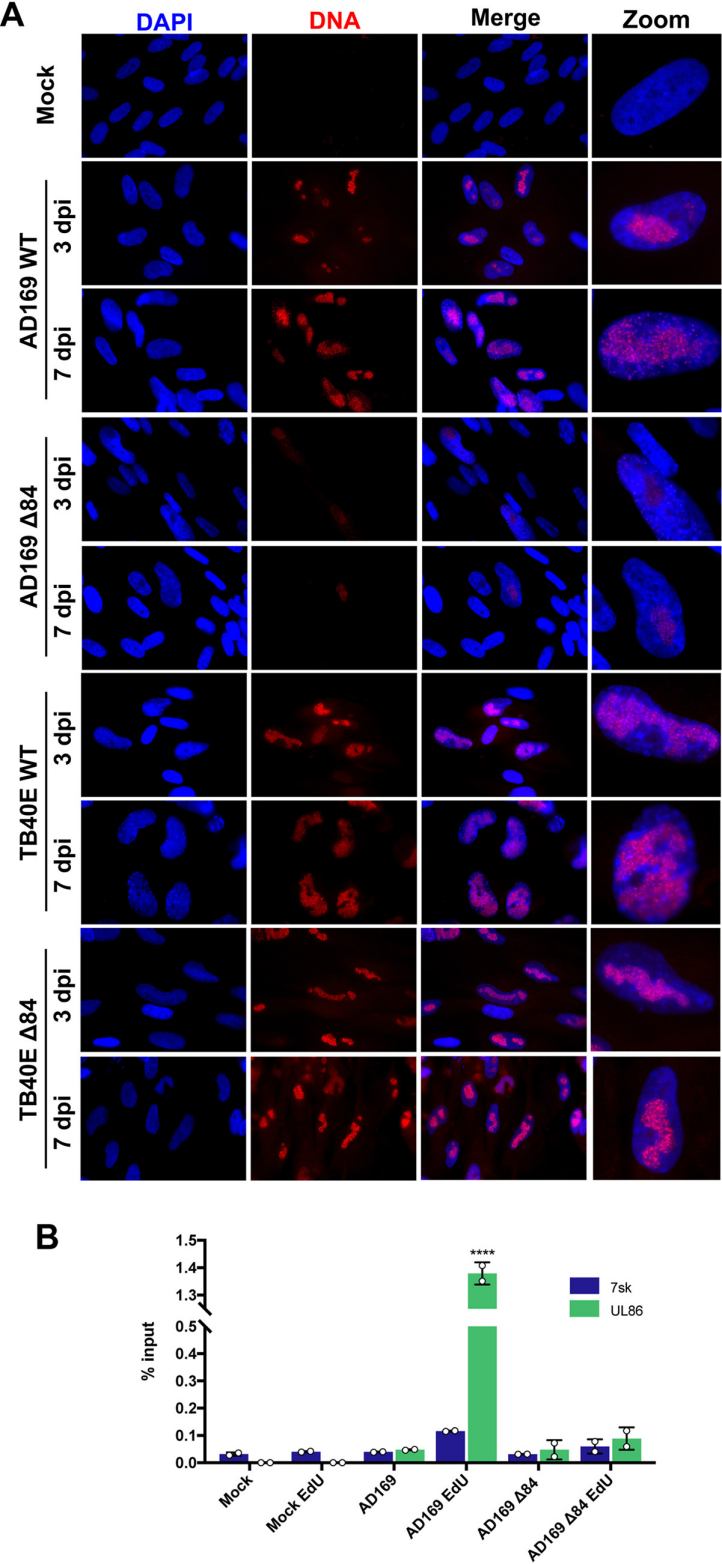
Viral DNA synthesis was decreased in cells infected with AD169 Δ84. (A) HF cells were infected (MOI = 4) with AD169 WT, AD169 Δ84, TB40E WT, or TB40E Δ84. After 3 dpi and 7 dpi, cells were incubated with 10 μm EdU for 30 min for incorporation into replicating DNA. EdU-labeled DNA was conjugated to Alexa Fluor 594 (red) by click chemistry. Nuclei were stained with DAPI (blue). Cells were imaged at ×63 magnification. (B) HF cells were either mock infected or infected with AD169 WT or AD169 Δ84. Cells were labeled with EdU (30 min) after 2 dpi. FENDI experiments were performed to isolate EdU-labeled DNA. Enriched DNA was quantified by qPCR and percent input was calculated. Error bars represent standard deviation (SD) of two independent experiments (*n* = 2). Statistical analysis was performed using two-way ANOVA; ****, *P* < 0.0001.

Considering that UL84 and IE2 are proposed to be the initiation factors or OBPs required for *ori*Lyt-dependent DNA replication, defects in viral DNA synthesis were explored. Nascent DNA replication was visualized using 5-ethynyl-2-deoxyuridine (EdU) labeling techniques (Fig. 2A). EdU is a thymidine analog that is incorporated into replicating DNA. This molecule harbors an alkyne group that can undergo a “click” reaction and be conjugated to biomolecules, such as a fluorescent dye or biotin. In these EdU labeling techniques, the state of HF confluence is crucial, as it is necessary to limit labeling of cellular DNA. Superconfluent HF cells undergo contact inhibition and will cease proliferation and cellular DNA replication. This allows for specific labeling of viral DNA versus cellular DNA *in vitro*. In our previous work, we optimized the labeling conditions for HCMV infections to capture as much viral DNA synthesis while reducing the amount of labeled cellular DNA (26).

Viral DNA replication was visualized by adding prewarmed media containing EdU to superconfluent HF cells that were infected with wild-type (WT) and Δ84 mutants of either AD169 or TB40E strains. Viral DNA was labeled with EdU for 30 min followed by a click reaction to conjugate 594-fluorophore (red) to the newly synthesized and labeled DNA. Imaging of EdU-labeled DNA was performed at 3 and 7 dpi (Fig. 2A). HF cells infected with AD169 WT, TB40E WT, and TB40E Δ84 displayed labeled DNA, indicating ongoing viral DNA synthesis during the incubation with EdU. The aggregating pattern of EdU-labeled DNA is consistent with viral replication compartments within the host cell nucleus. In contrast, for cells infected with the AD169 Δ84 mutant, it was difficult to visually discern any labeled DNA and a very small amount of fluorescence was observed within the nucleus. It is important to note that the faint detection of EdU-labeled DNA in AD169 Δ84 may be due to the presence of a small amount of UL84 mRNA which could potentially be packaged in the virion and carried over after being propagated and harvested from the T-HF 84 cell line. This speculation is based on previous reports which have demonstrated that viral mRNA is packaged in the virion (27–29). Another possibility could be unproductive initiation of DNA synthesis, creating short regions with EdU incorporation but not accumulating to the levels of WT. The exact mechanism of initiation is unknown but suggested to involve promoter activity within *ori*Lyt, unwinding and disruption of the Y-block, and recruitment of the core enzymatic replication machinery. There remains the possibility that AD169 can perform some of these actions in the absence of UL84, resulting in limited DNA synthesis and EdU incorporation that can be faintly detected, but cannot complete efficient DNA replication without UL84. The activity of the *ori*Lyt promoter and localization of replication protein, such as UL44, in TB40E and AD169 is explored in experiments detailed below.

The deficiency of viral DNA synthesis in AD169 Δ84 was quantified by fast and efficient nascent DNA isolation (FENDI) (26). In the FENDI method, newly synthesized DNA is labeled with EdU and conjugated to biotin. The nascent DNA is isolated and purified by streptavidin bead capture, followed by quantitative PCR (qPCR) analysis. In these experiments, HF cells were either mock infected or infected with AD169 WT or AD169 Δ84 (Fig. 2B). Newly synthesized, EdU-labeled DNA was isolated and analyzed by qPCR using primers and probes specific for a viral gene (UL86) and cellular gene (7SK). Similar to what was observed in the EdU-labeled DNA imaging, AD169 WT replication showed significant EdU incorporation indicative of active viral DNA synthesis. For AD169 Δ84, there was no measurable DNA synthesis, affirming the requirement of UL84 for DNA synthesis. Additionally, there was no significant enrichment for cellular DNA, suggesting that the EdU-labeled DNA in the imaging is predominately viral (Fig. 2A). These data verify that UL84 is a required factor for viral DNA replication in UL84-dependent strains.

### Characterizing UL84 and IE2 proteins in HCMV strains.

**FIG 3:**
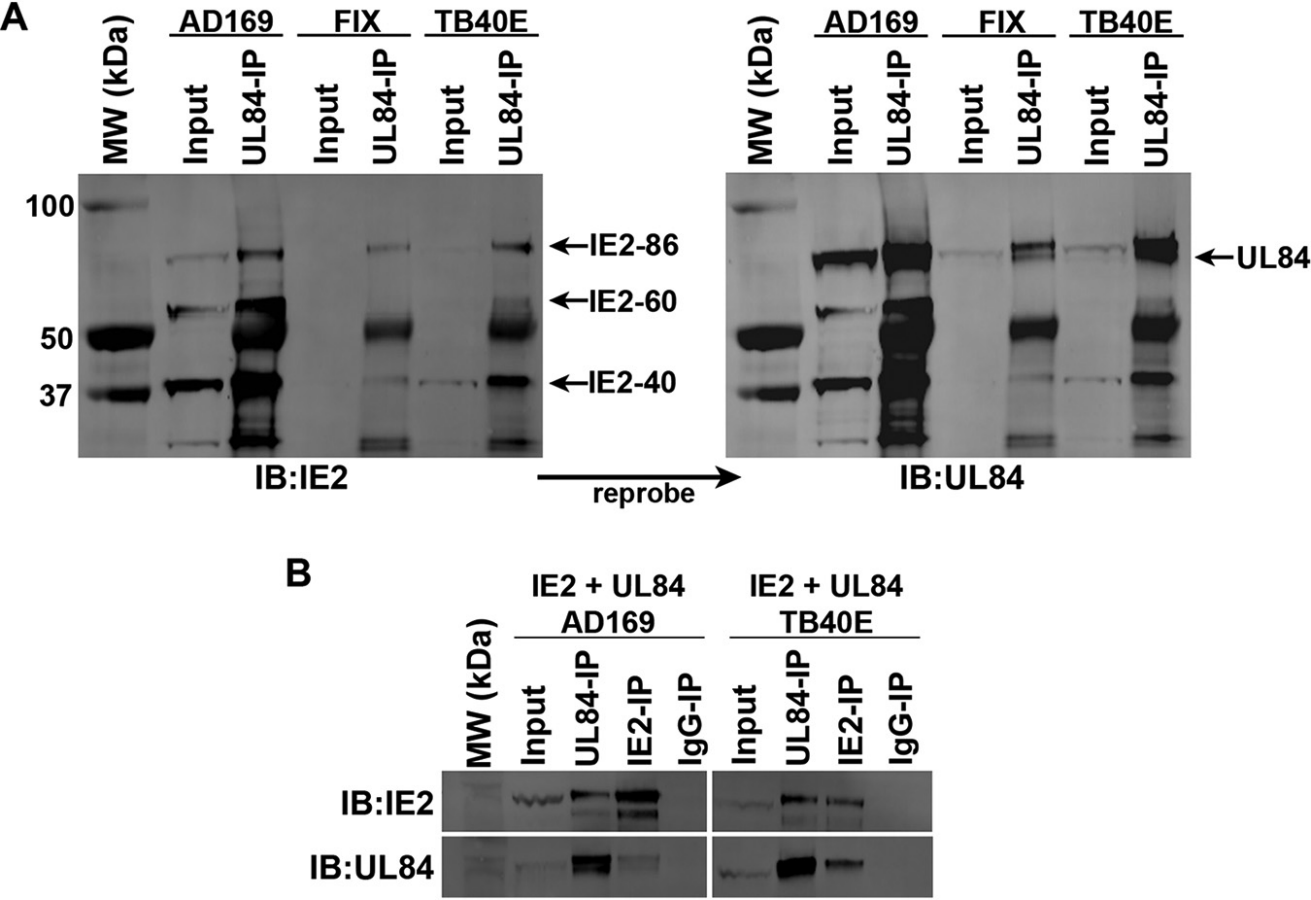
Viral proteins UL84 and IE2 interacted in both UL84-dependent and UL84-independent strains of HCMV. (A) HF cells were infected with AD169, FIX, or TB40E (MOI = 1). After 6 dpi, cell lysate was harvested and a UL84-IP was performed. Protein was resolved by SDS-PAGE and transferred to a polyvinylidene difluoride (PVDF) membrane. Membrane was probed with IE2 antibody followed by reprobing with UL84 antibody. (B) 293FT cells were cotransfected with UL84 and IE2 plasmids (AD169 or TB40E) as indicated. Co-IPs were performed with antibodies for UL84, IE2, or an IgG negative control. Protein was resolved by SDS-PAGE and transferred to a PVDF membrane. Membrane was probed with IE2 or UL84 antibody as indicated.

The interaction of UL84 and IE2 during an infection has been well characterized (18, 30, 31). Therefore, we evaluated whether there was a difference in the protein interaction of IE2 and UL84 between UL84-dependent and UL84-independent strains. HF cells were infected with AD169, FIX (UL84-dependent), or TB40E. Infections were harvested after 6 dpi, and coimmunoprecipitations (co-IPs) were performed using a UL84 specific antibody (Fig. 3A). In all strains, the IE2 protein was detected in the UL84-IPs, indicating no discernible difference in the interaction between UL84 and IE2. Also, all IE2 protein products were detected to interact with UL84, including IE2-86, IE2-60, and IE2-40.

To further characterize the protein-protein interaction and assess if there were additional viral factors present in infection which were contributing to the interaction between UL84 and IE2, we constructed expression plasmids using pSI vector (containing the SV40 promoter) and cloned in the UL84 (pSI-UL84) and IE2 (pSI-IE2) gene sequences from either AD169 or TB40E. The expression plasmids were transfected into 293FT cells, followed by co-IP using UL84 or IE2 specific antibody (Fig. 3B). By Western blotting, we detected IE2 protein in the UL84-IPs and UL84 protein in IE2-IPs among both AD169 and TB40E strains. Co-IPs performed in both HCMV-infected cells and cells transfected with IE2 and UL84 expression plasmids showed interactions for IE2 and UL84 from TB40E similar to what has previously been described for AD169 (18, 32).

**FIG 4:**
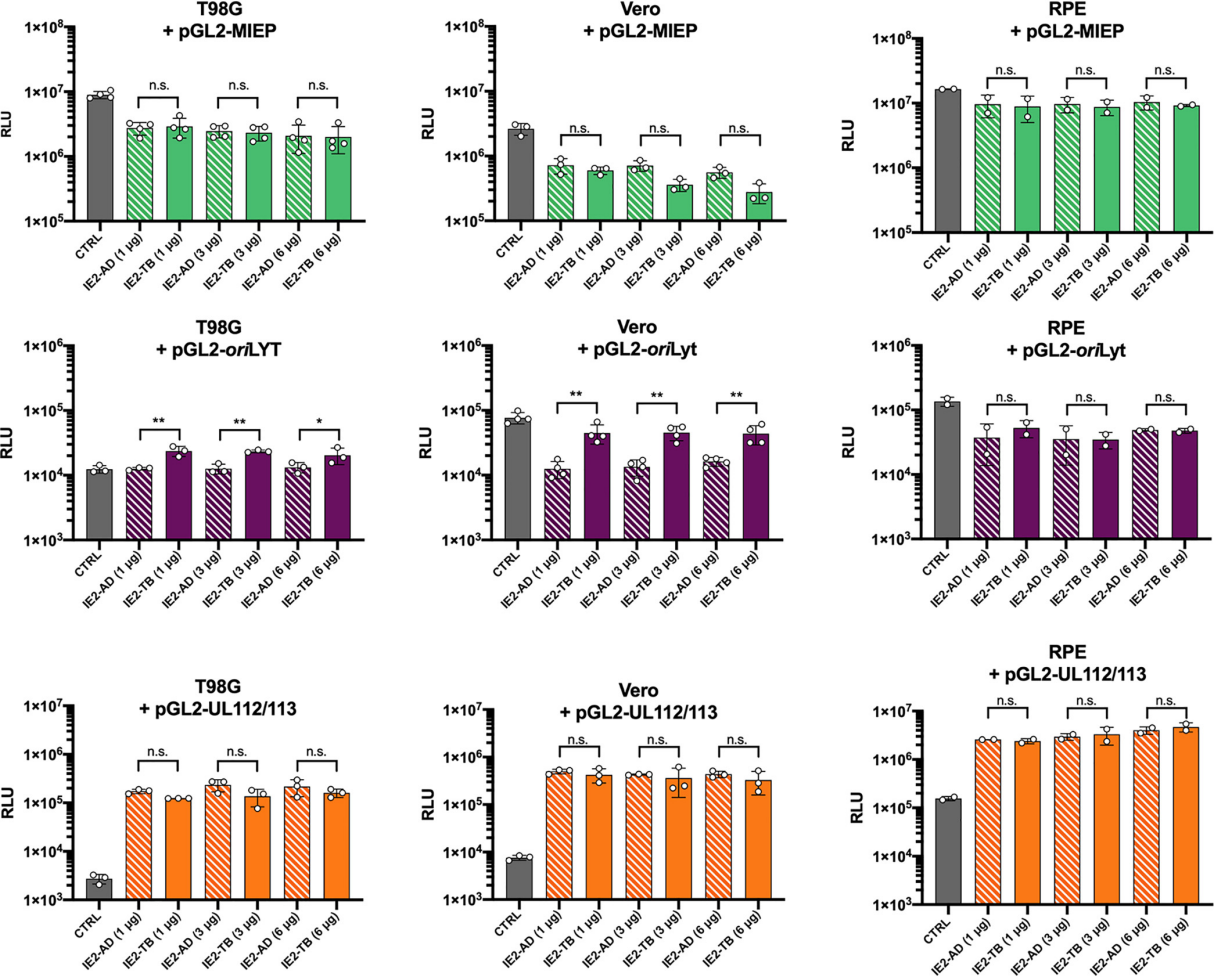
Bar charts comparing activity of *ori*Lyt, MIE, and UL112/113 promoters in the presence of IE2 from AD169 or TB40E. T98G, Vero, and RPE cells were transfected with luciferase reporter constructs containing the viral promoters pGL2-MIEP, pGL2-*ori*Lyt, and pGL2-UL112/113 alone (CTRL) or with pSI-IE2 constructed from either strain AD169 or strain TB40E (IE2-AD or IE2-TB) at a range of 1 to 6 μg, as indicated. Cells were harvested 48 hpt and luciferase assay was performed. Error bars indicate SD from independent experiments (*n* = 2 to 4). Statistical analysis was performed using one-way ANOVA. *, *P *< 0.05; **, *P *< 0.005; n.s., not significant.

As there was no appreciable difference in UL84 and IE2 protein interactions among strains, we tested for any changes in transactivation function of IE2 at viral promoters by luciferase assay (Fig. 4). One of the documented functions of UL84 from AD169 is to relieve the repression of IE2 at the MIE and *ori*Lyt promoter (16, 33, 34). If UL84 is not required in TB40E, this brings into question whether or not IE2 (H388D) still has the ability to repress the MIE and *ori*Lyt promoter. We performed luciferase assays using the MIE, *ori*Lyt, and UL112/113 promoters cloned into luciferase reporter plasmids (pGL2), along with plasmids expressing either AD169 IE2 or TB40E IE2 (H388D). Our previously discussed data were performed primarily in HF cells. However, with these primary cells it is difficult to achieve adequate transfection efficiency. To address this issue, we performed transfection and luciferase experiments in three cell lines permissible to HCMV infection, T98G, Vero, and retinal pigmented epithelium (RPE) cells.

The initial experiments were performed by transfecting T98G, Vero, and RPE cells with the luciferase reporter plasmid (pGL2) containing HCMV promoter sequences (MIE promoter [MIEP], *ori*Lyt, and UL112/113) alone or along with pSI-IE2 (IE2-AD for AD169 or IE2-TB for TB40E) at increasing concentrations (Fig. 4). For each concentration of IE2, the average relative luciferase unit (RLU) of the replicates was used to compare promoter activity in the presence of IE2 from AD169 (IE2-AD) and IE2 from TB40E (IE2-TB) using a one-way analysis of variance (ANOVA). There was a similar amount of repression comparing IE2 from TB40E and IE2 from AD169 for the MIE promoter (MIEP) in all cell lines tested. In contrast, for the *ori*Lyt promoter, TB40E IE2 in Vero cells showed less repression, or in the case of T98G cells showed slight activation, and was significantly different compared to AD169 IE2. In RPE cells, the difference in the *ori*Lyt promoter activity between IE2-TB and IE2-AD was not statistically significant. The UL112/113 promoter was used as a representative early viral promoter and in these luciferase assays showed comparable amounts of activity, and there was no significant difference between IE2-AD and IE2-TB.

**FIG 5:**
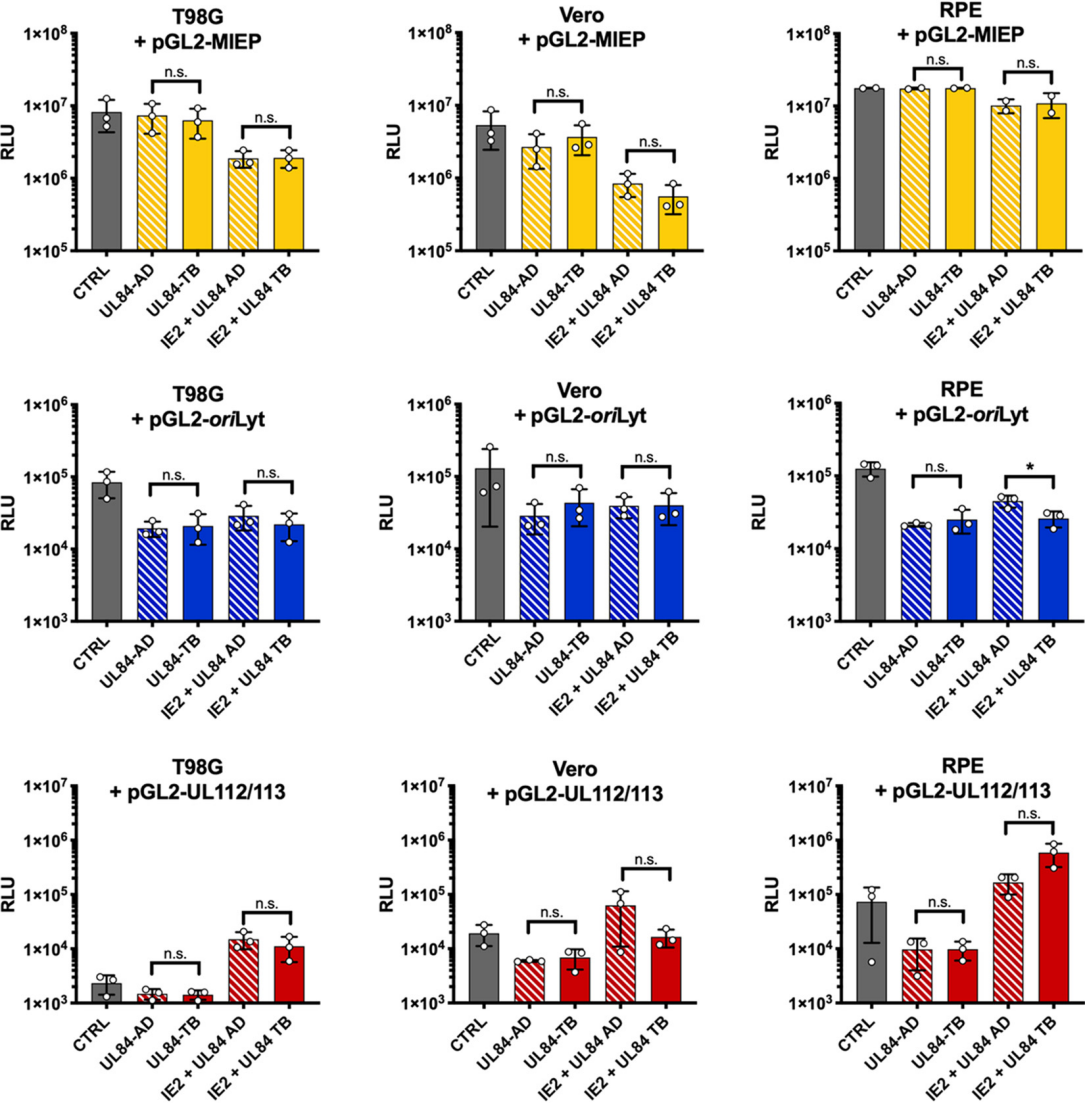
Bar charts comparing activity of oriLyt, MIE, and UL112/113 promoters with UL84 and IE2 from AD169 or TB40E. T98G, Vero, and RPE cells were cotransfected with luciferase reporter constructs containing the following viral promoters: pGL2-MIEP, pGL2-*ori*Lyt, and pGL2-UL112/113 alone (CTRL) or with pSI-UL84 or pSI-IE2 from AD169 or TB40E (IE2 + UL84 AD, UL84-AD, IE2 + UL84 TB, UL84-TB). Cells were harvested after 48 hpt and a luciferase assay was performed. Error bars indicate SD from independent experiments (*n* = 2 to 3). Statistical analysis was performed using a *t*-test. *, *P *< 0.05; n.s., not significant.

During an infection, UL84 interacts with IE2 and consequently regulates the transcriptional activity of IE2. At the *ori*Lyt promoter, UL84 is known to mitigate the repressive activity of IE2 (16). Similarly, previous reports have demonstrated that UL84 can relieve IE2 repression at MIEP, while at the UL112/113 promoter UL84 can inhibit the transactivation by IE2 (33). To investigate whether these IE2 and UL84 interactions affect promoter activity differently between strains, we cotransfected UL84 alone or along with IE2 (Fig. 5). Statistical analysis was used to assess differences in the promoter activity in the presence of UL84-AD and UL84-TB alone or IE2 plus UL84-AD and IE2 plus UL84-TB. In the presence of UL84 alone, there was no statistical difference between UL84-AD and UL84-TB with any of the promoters tested. Comparing the differences in the ability of UL84 to regulate IE2 transcriptional activity, only the *ori*Lyt promoter in RPE cells was found to have a significant difference between AD169 and TB40E. For UL84 and IE2 from TB40E, there was less relief of the IE2-mediated repression at *ori*Lyt promoter by UL84 than for those from AD169. It is also notable that in RPE cells there was an increase in the UL112/113 promoter activity in the presence of UL84 from TB40E, although it did not reach the level of statistical significance. These data suggest that there may be a difference in the ability of UL84 from TB40E to regulate the transcriptional activity of IE2, and it may be due to cellular factors present in specific cell types, as seen with RPE cells.

### Generation and phenotype of TB40E ΔUL84 IE2 D388H.

**FIG 6:**
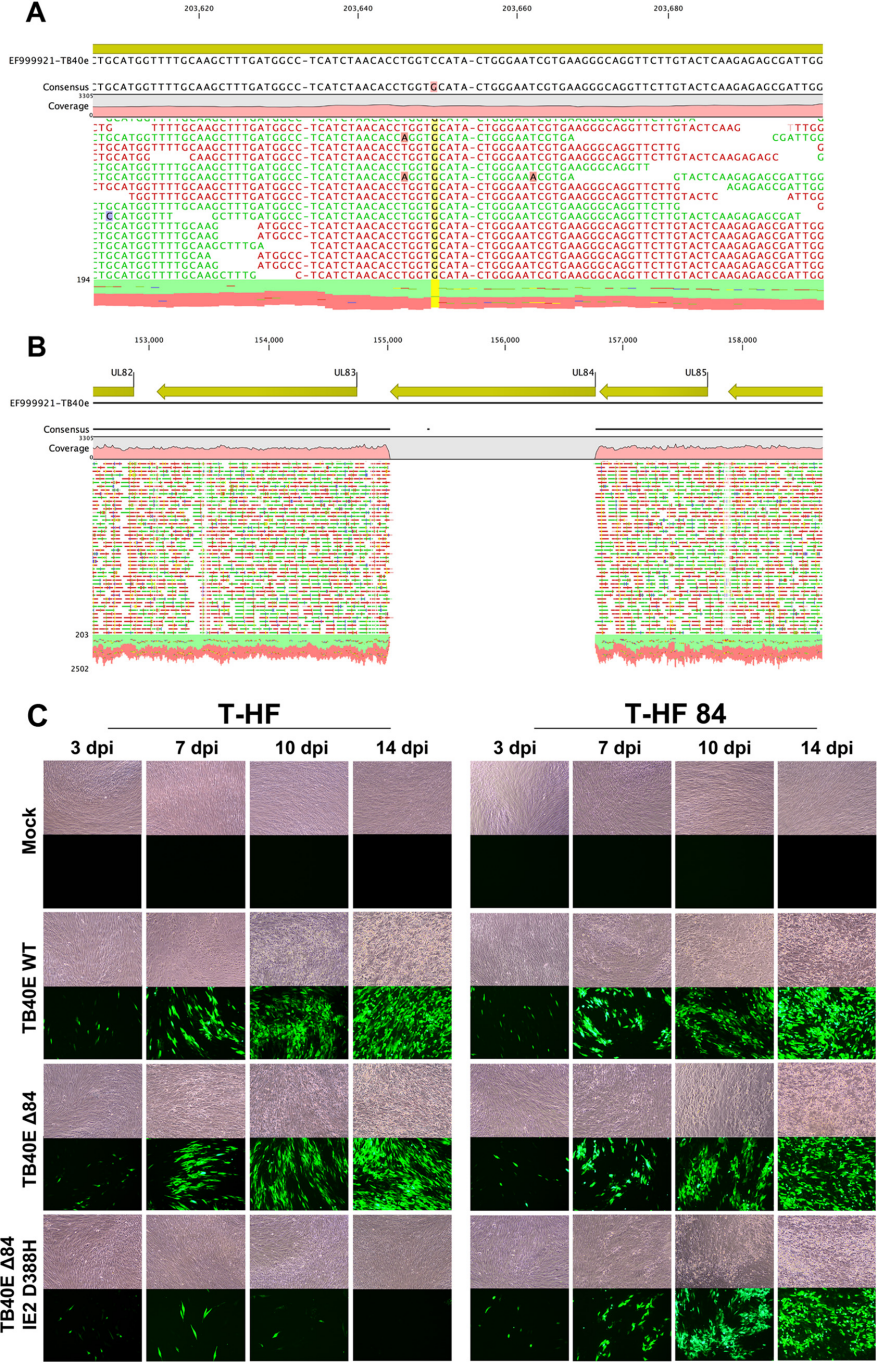
TB40E Δ84 IE2 D388H mutant virus replicated and spread on T-HF 84 cell line. The TB40E Δ84 IE2 D388H was generated using BAC recombineering. Whole-genome sequencing was performed to confirm IE2 and UL84 mutations. Qiagen CLC genomics workbench was used to map the reads to the HCMV reference genome. (A) Mapping of TB40E Δ84 IE2 D388H mutant to the reference genome, at IE2 (UL122) locus. Yellow highlight shows the single nucleotide change that produced a transcript which resulted in a single amino acid switch during translation (IE2 D388H). (B) Mapping of TB40E Δ84 IE2 D388H to the reference genome showing the UL84 deletion. (C) T-HF and T-HF 84 cells were either mock infected or infected with TB40E WT, TB40E Δ84, or TB40E Δ84 IE2 D388H (MOI = 1). Light and fluorescence microscopy images were taken at 3 dpi, 7 dpi, 10 dpi, and 14 dpi. Images were taken at ×10 magnification.

To evaluate if other viral factors besides IE2 (H388D) were contributing to the ability of some HCMV strains to replicate without UL84, we generated a TB40E ΔUL84 IE2 D388H mutant. BAC mutagenesis using WT TB40E was used to first introduce the amino acid switch of IE2. For this recombineering method, a double-stranded DNA gBlock (IDT) was designed to include a single nucleotide switch in the UL122 locus. The genotypic alteration was originally 5′-GTC-3′ changed to 5′-GTG-3′ on the coding strand (Fig. 6A), since IE2 is transcribed on the complementary strand of DNA from the 5′-CAC-3′ sequence which corresponds to the aspartic amino acid in place of 5′-GAC-3′, a histidine codon. After the TB40E IE2 D388H was made, the UL84 locus was removed to produce the TB40E ΔUL84 IE2 D388H mutant. All genetic mutations were verified by whole-genome next-generation sequencing (Fig. 6B).

The growth phenotype of the TB40E ΔUL84 IE2 D388H mutant compared to that of TB40E WT was tested by infecting T-HF and T-HF UL84 cells (Fig. 6C). T-HF and T-HF 84 cells were infected (MOI = 1) and imaging was performed at 3, 7, 10, and 14 dpi. At 3 dpi, all infections showed GFP+ cells, indicating a successful initial infection. For the TB40E ΔUL84 IE2 D388H mutant virus in T-HF cells, there was noticeably no viral spread by 10 and 14 dpi, and the initial GFP signal was almost completely abolished at 14 dpi. Infection of TB40E ΔUL84 IE2 D388H on the T-HF 84 cell line rescued viral production, and viral spread was observed at the later time points. This indicated that by altering the IE2 H388D sequence in the UL84-independent strain TB40E to IE2 D388H, the virus was replication defective in the absence of UL84. IE2 therefore remains the single factor responsible for preserving replication in UL84-independent strains.

**FIG 7:**
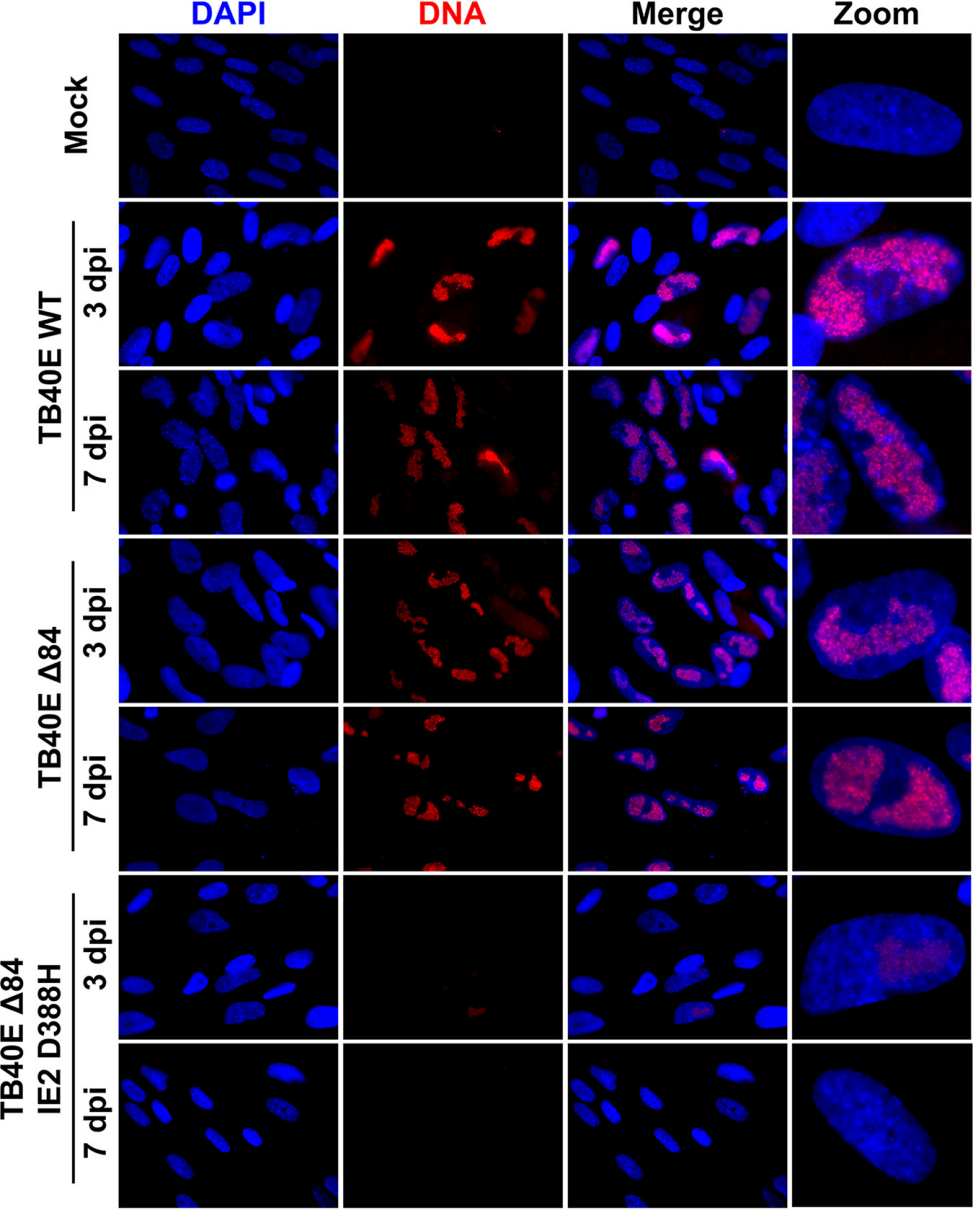
Viral DNA synthesis was decreased in cells infected with TB40E Δ84 IE2 D388H. HF cells were infected (MOI = 4) with TB40E WT, TB40E Δ84, or TB40E Δ84 IE2 D388H. After 3 dpi or 7 dpi, cells were incubated with 10 μm EdU for 30 min to label replicating DNA. EdU-labeled DNA was conjugated to Alexa Fluor 594 (red) by click chemistry. Nuclei were stained with DAPI (blue). Cells were imaged at ×63 magnification.

Additional imaging experiments were performed to evaluate the ability of TB40E ΔUL84 IE2 D388H to incorporate the nucleoside analogue EdU, similar to previous experiments performed with AD169 ΔUL84 (Fig. 2). Confluent HF cells were infected with TB40E WT, TB40E ΔUL84, or TB40E ΔUL84 IE2 D388H, and cells were pulsed with 10 μM EdU at 3 and 7 dpi. For the UL84-independent strains, TB40E WT and TB40E ΔUL84, there was visible accumulation of EdU-labeled DNA in replication compartments within the nucleus. In contrast, for TB40E ΔUL84 IE2 D388H, there was very slight incorporation at 3 dpi, similar to what was observed with AD169 ΔUL84 (Fig. 2), and by 7 dpi there was no visible incorporation of EdU. These data, taken together with the lack of viral spread following infection (Fig. 7), suggest that the TB40E ΔUL84 IE2 D388H is unable to replicate due to a defect in viral DNA synthesis.

### Protein localization in UL84-dependent and UL84-independent strains.

**FIG 8:**
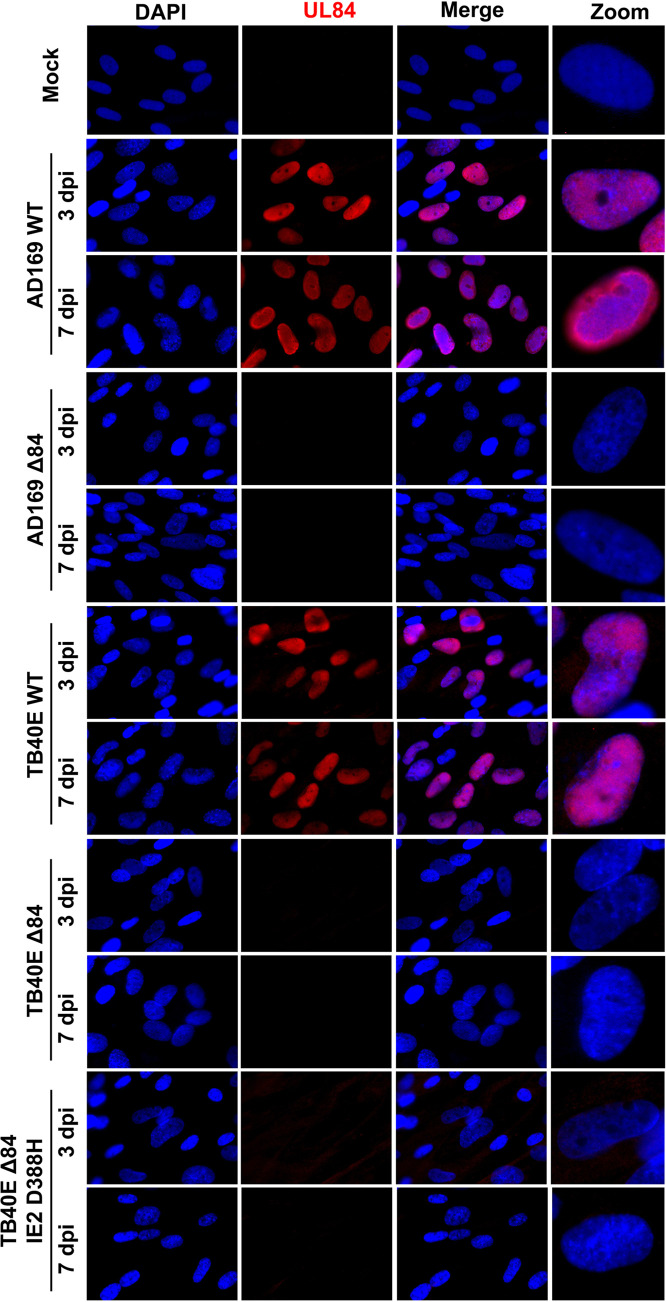
UL84 was not detected in Δ84 HCMV mutant viruses by IFA. HF cells were mock infected or infected with AD169 WT, AD169 Δ84, TB40E WT, TB40E Δ84, or TB40E Δ84 IE2 D388H as indicated (MOI = 4). After 3 dpi and 7 dpi, IFA was performed to detect UL84 protein expression (red). Nuclei were stained with DAPI (blue). Images were taken at ×63 magnification.

**FIG 9:**
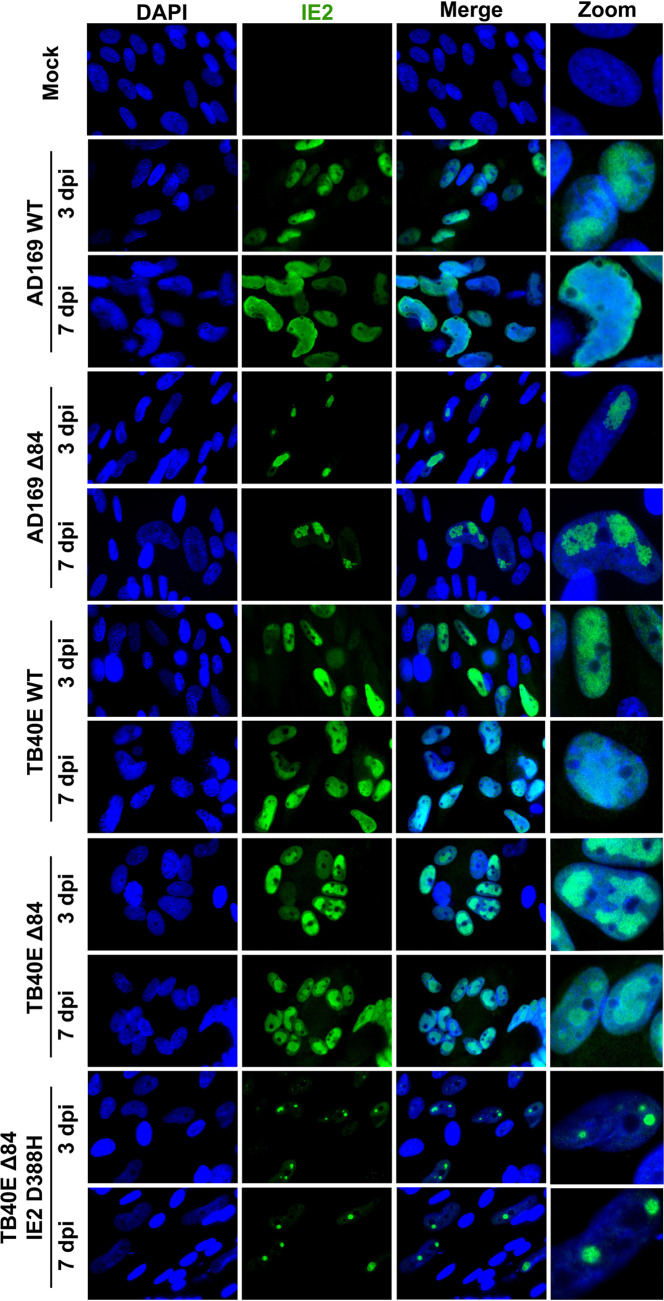
IE2 localization patterns in UL84-independent and UL84-dependent HCMV strains. HF cells were mock infected or infected with AD169 WT, AD169 Δ84, TB40E WT, TB40E Δ84, or TB40E Δ84 IE2 D388H as indicated (MOI = 4). After 3 and 7 dpi, IFA was performed to detect IE2 (green). Nuclei were stained with DAPI (blue). Images were taken at ×63 magnification.

Next, we evaluated whether there were localization differences of IE2 and UL84 between strains. HF cells were infected with AD169 WT, AD169 Δ84, TB40E WT, TB40E Δ84, or TB40E ΔUL84 IE2 D388H or were mock infected. At 3 and 7 dpi, cells were fixed and permeabilized, followed by the addition of specific primary antibodies to detect UL84 and IE2, and secondary antibody was conjugated to Alexa Fluor for visualization using a fluorescence microscope. UL84 protein was detected only in WT infections and not in cells infected with Δ84 mutants (Fig. 8). IE2 protein expression was observed in all WT and Δ84 mutants (Fig. 9). The pattern of IE2 was typically dispersed throughout the host cell nucleus as seen in AD169 WT, TB40E WT, and TB40E Δ84. Interestingly, the IE2 expression detected in an AD169 Δ84 and TB40E ΔUL84 IE2 D388H, which harbors the same IE2 sequence as AD169, remained concentrated in a punctate formation within the nucleus.

**FIG 10:**
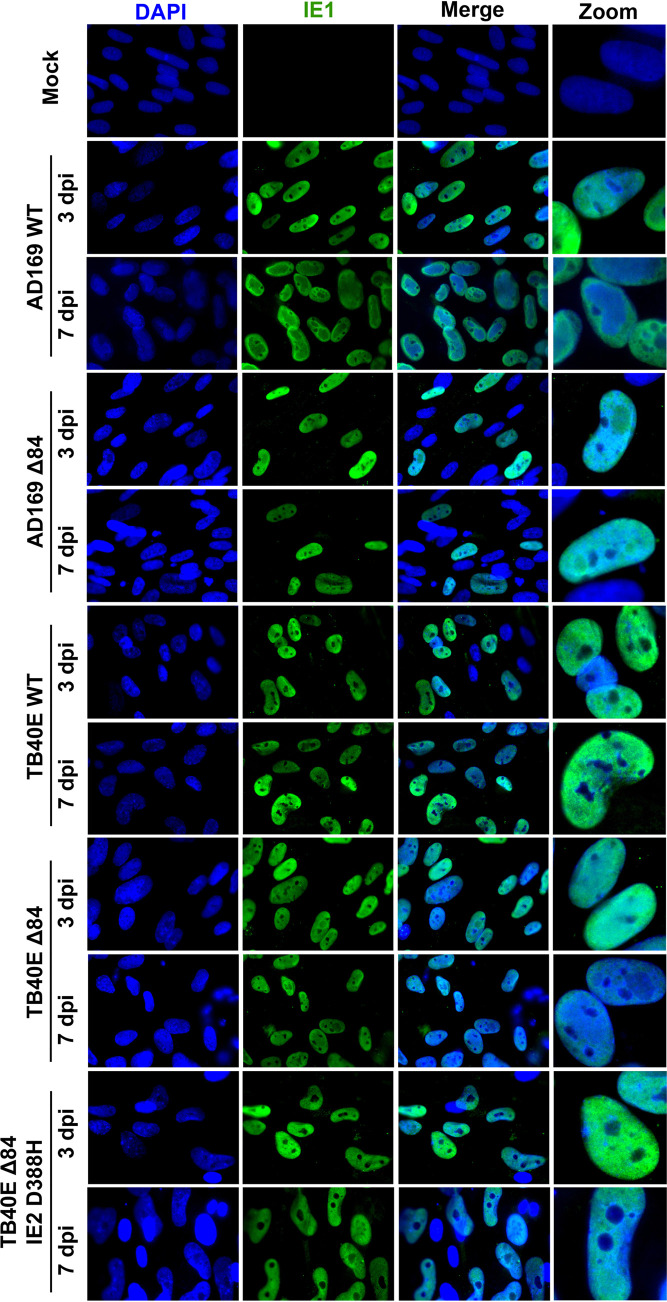
IE1 protein localization in UL84-independent and UL84-dependent HCMV strains. HF cells were mock infected or infected with AD169 WT, AD169 Δ84, TB40E WT, TB40E Δ84, or TB40E Δ84 IE2 D388H as indicated (MOI = 4). After 3 dpi and 7 dpi, IFA was performed to detect IE1 (green). Nuclei were stained with DAPI (blue). Images were taken at ×63 magnification.

**FIG 11:**
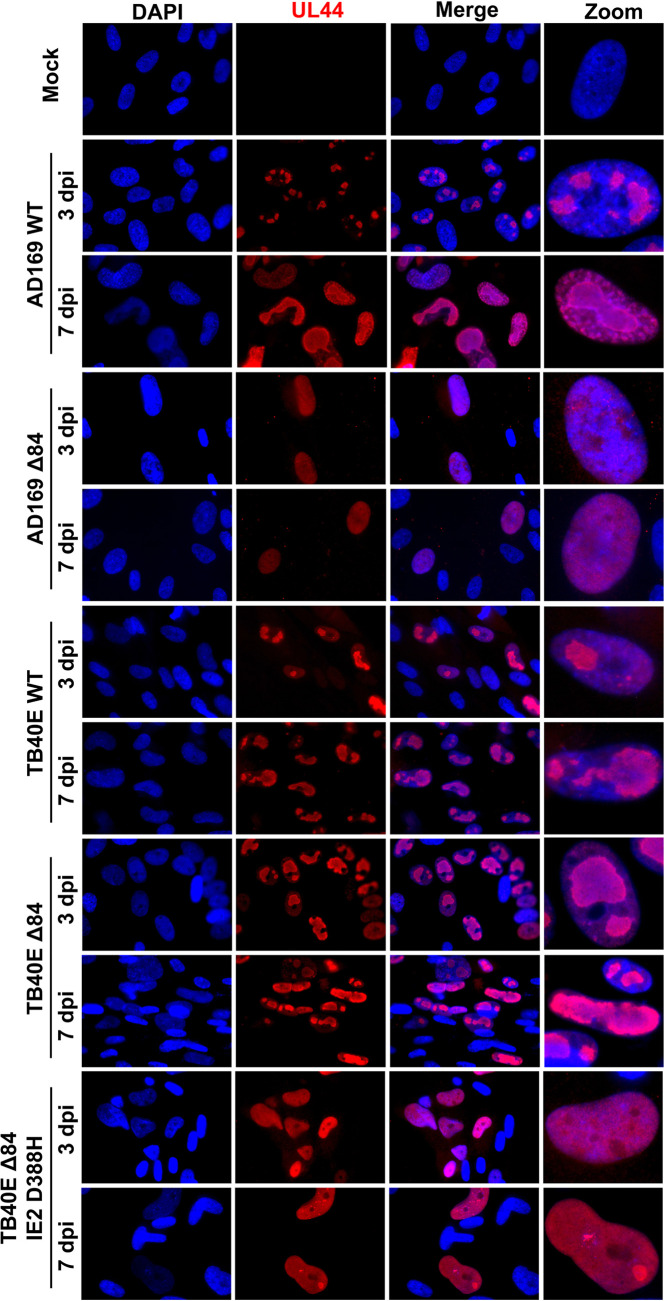
UL44 localization patterns in UL84-independent and UL84-dependent HCMV strains. HF cells were mock infected or infected with AD169 WT, AD169 Δ84, TB40E WT, TB40E Δ84, or TB40E Δ84 IE2 D388H as indicated (MOI = 4). After 3 dpi and 7 dpi, IFA was performed to detect UL44 (red). Nuclei were stained with DAPI (blue). Images were taken at ×63 magnification.

The localization of two other viral proteins, IE1 and UL44, was assessed by immunofluorescence assay (IFA) image in cells infected with AD169 WT, AD169 Δ84, TB40E WT, TB40E Δ84, or TB40E ΔUL84 IE2 D388H at 3 and 7 dpi. IE1 was chosen because it originates from the same genomic locus as IE2. IE1 and IE2 share exons 2 and 3 but are differentially spliced to contain exon 4 in IE1 and exon 5 in IE2 (35). UL44 is the viral polymerase processivity factor and has previously been reported to interact with both UL84 and IE2 (31, 36–38). For the IFA of IE1, there was no discernible difference in the localization pattern between AD169 or TB40E in WT and Δ84 mutants (Fig. 10). In the IFA of UL44, there was a slightly different nuclear localization pattern of UL44 in those viruses which are able to synthesize DNA (AD169 WT, TB40E WT, and TB40E Δ84) and those that are unable to efficiently synthesize DNA (AD169 Δ84 and TB40E ΔUL84 IE2 D388H) (Fig. 11). UL44 is required for viral DNA synthesis and predominately localizes to the replication compartments within the nucleus, as seen in AD169 WT, TB40E WT, and TB40E Δ84. In the virus mutants AD169 Δ84 and TB40E ΔUL84 IE2 D388H, with a defect in DNA synthesis, UL44 has a more diffuse localization within the nucleus without the formation of distinct replication compartments.

### ChIP-seq analysis of IE2 and UL84 in AD169 and TB40E.

**FIG 12:**
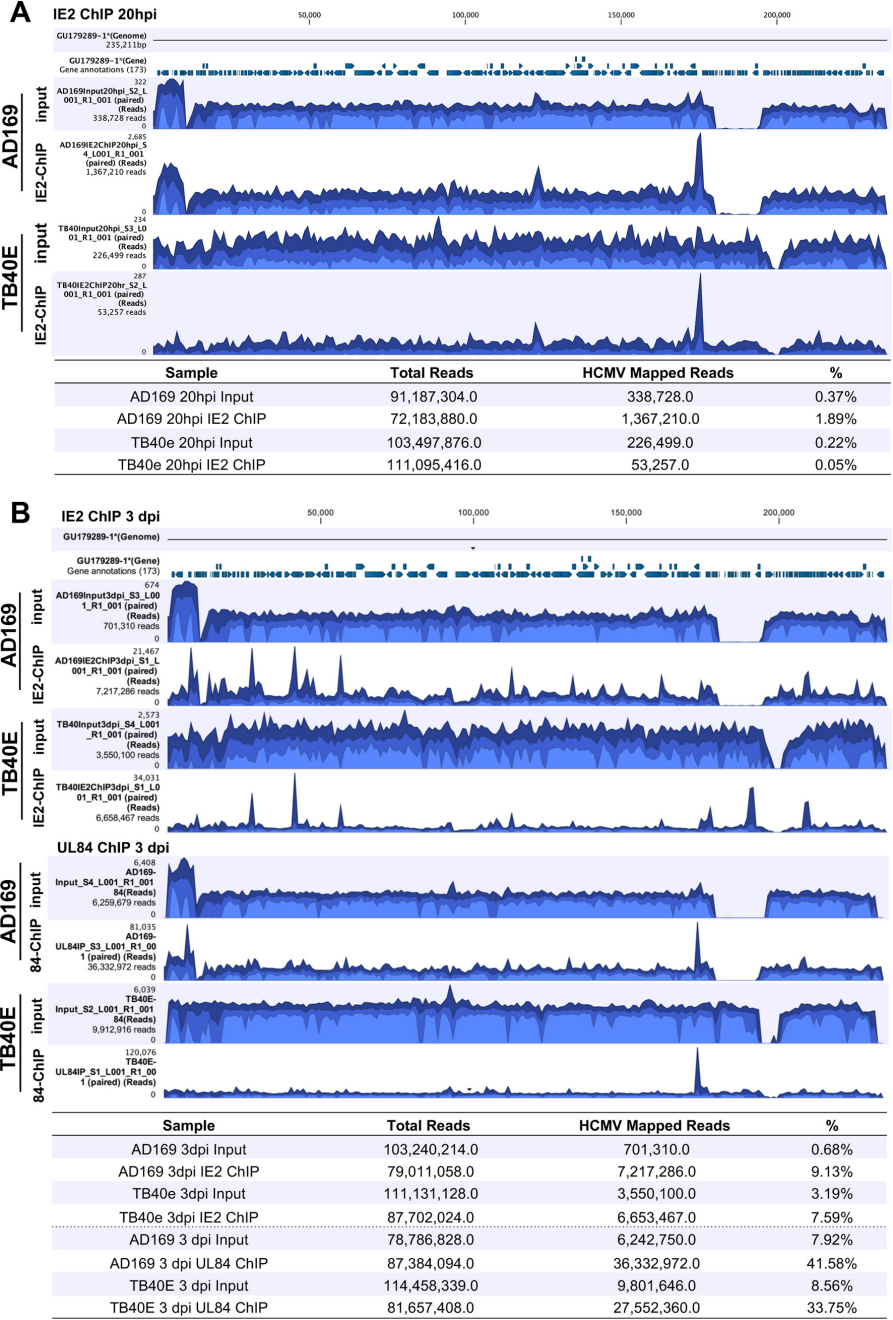
Mapped reads and alignment to the viral genome from IE2 and UL84 ChIP-seq. HF cells were infected with AD169 and TB40E (MOI = 4). Cells were harvested at (A) 20 hpi for the IE2 ChIP or (B) 3 dpi for the IE2 and UL84 ChIP. Specific antibodies for UL84 or IE2 were used in immunoprecipitation as indicated. Libraries from input and ChIP samples were prepared and sequenced at the Nevada Genomic Center. ChIP-seq data analysis was performed using CLC genomics workbench to determine read alignment and generate images depicting coverage of the HCMV genome with areas of enrichment from the ChIP samples. Tables indicate the percentage of sequencing reads from input and ChIP samples that map to the reference HCMV genome.

Due to the unique and complex association of IE2 and UL84 at viral promoters, a ChIP-seq analysis was performed in order to determine if there were global changes to the enrichment of IE2 and UL84 on the viral genome. The ChIP-seq was carried out using antibodies to specifically target and enrich IE2 or UL84 along with associated DNA in both AD169 and TB40E strains to compare and identify unique binding interactions with the viral genome (Fig. 12). Since IE2 is an immediate-early transcript that is expressed within 2 to 4 h after infection, the IE2 ChIP was performed at 20 hpi and 3 dpi (Fig. 12A and B). UL84 is not abundantly detected until after 24 hpi; hence, the UL84-ChIP was performed only at 3 dpi during productive viral DNA synthesis (Fig. 12B). After the ChIP, sequencing libraries were prepared from input and ChIP DNA samples, followed by next-generation sequencing and analysis. IgG isotype antibodies were used along with the specific antibodies; because of the stringency in the protocol, the samples with the isotype controls did not have enough DNA to generate a sequencing library, so they were not processed further.

Input and ChIP sequencing reads were trimmed and aligned to the viral genome using CLC genomics workbench. Visualization of the read alignment across the entire viral genome show peak areas of enrichment for IE2 and UL84 (Fig. 12). The tables display total mapped reads and percentages of those reads specifically mapped to HCMV DNA. Interestingly, at 20 hpi the percentage of reads that mapped to HCMV DNA versus total reads in IE2-ChIP of AD169 was 1.89%, while in TB40E it was 0.05%. The total number of reads was lower in TB40E, suggesting that IE2 in TB40E is less enriched at the viral genome than is AD169 at early time points of infection. This feature may be due to the delayed onset of viral infection previously noted in TB40E (39). By 3 dpi, the percentage of mapped reads from IE2-ChIP was more comparable between AD169 at 9.13% and TB40E at 7.59%.

Another interesting feature identified was the high percentage of mapped HCMV reads in the UL84 ChIP. In AD169, 41.58% of UL84 ChIP reads were mapped to the viral genome, and for TB40E it was 33.75%. Comparing IE2 ChIP and UL84 ChIP, UL84 interacts with the viral genome more exclusively than does IE2. As less than 10% of IE2-ChIP reads mapped to the viral genome, we hypothesize that this may be due to the dual nature of IE2 interacting with both the viral and the cellular genomes.

**FIG 13:**
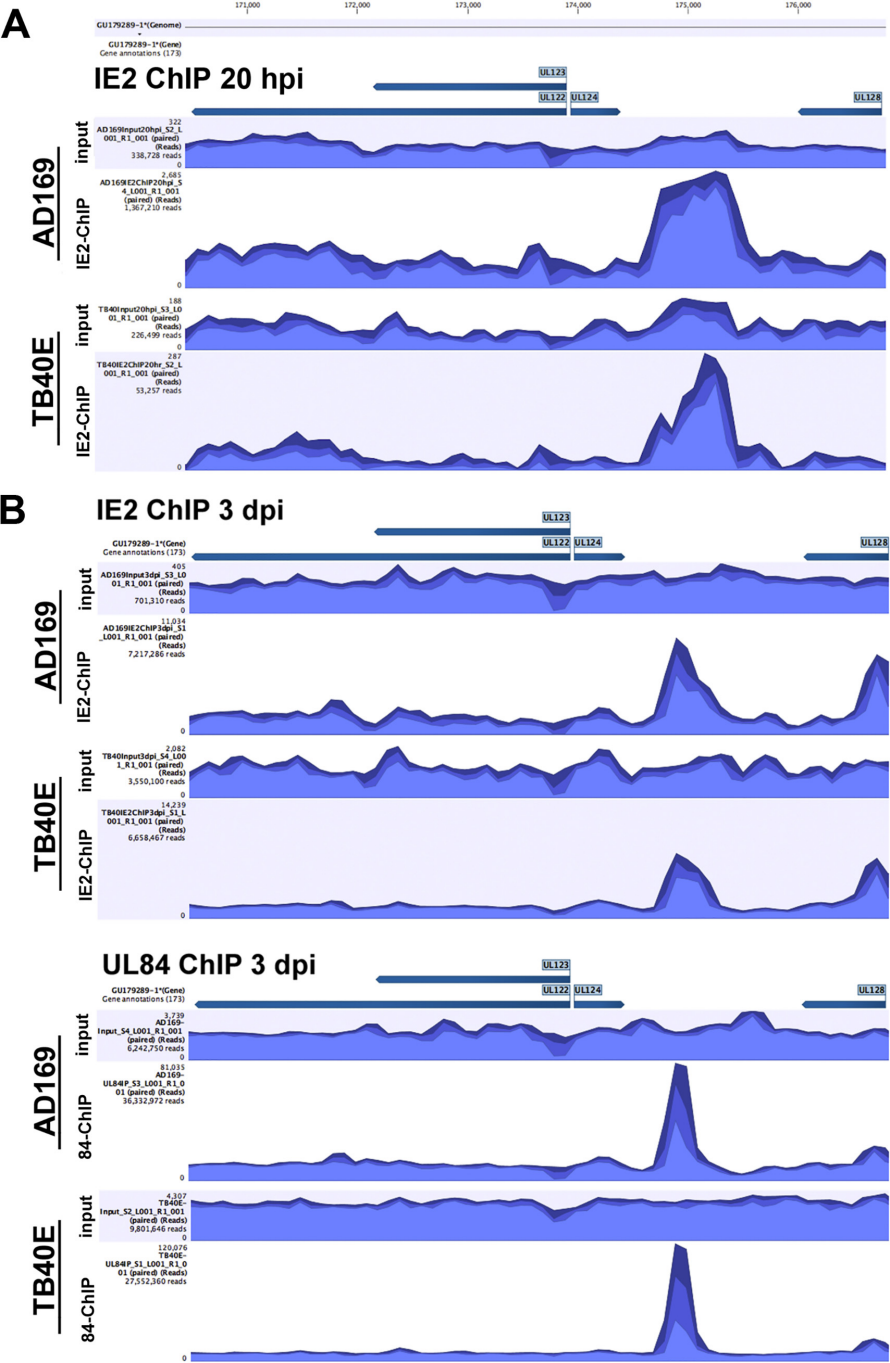
Mapping of IE2 and UL84 ChIP-seq to MIE genomic locus in AD169 and TB40E. The enrichment of UL84 or IE2 to the MIE genomic region in AD169 and TB40E was determined by ChIP-seq analysis. Reads from input and ChIP libraries were aligned to the viral genome and visualized using CLC genomics workbench. Sequencing aligned to the MIE region, encompassing UL122/123 and the promoter region upstream of the open reading frame, is depicted for input and ChIP samples at (A) 20 hpi for the IE2 ChIP and (B) 3 dpi for the IE2 and UL84 ChIP.

**FIG 14:**
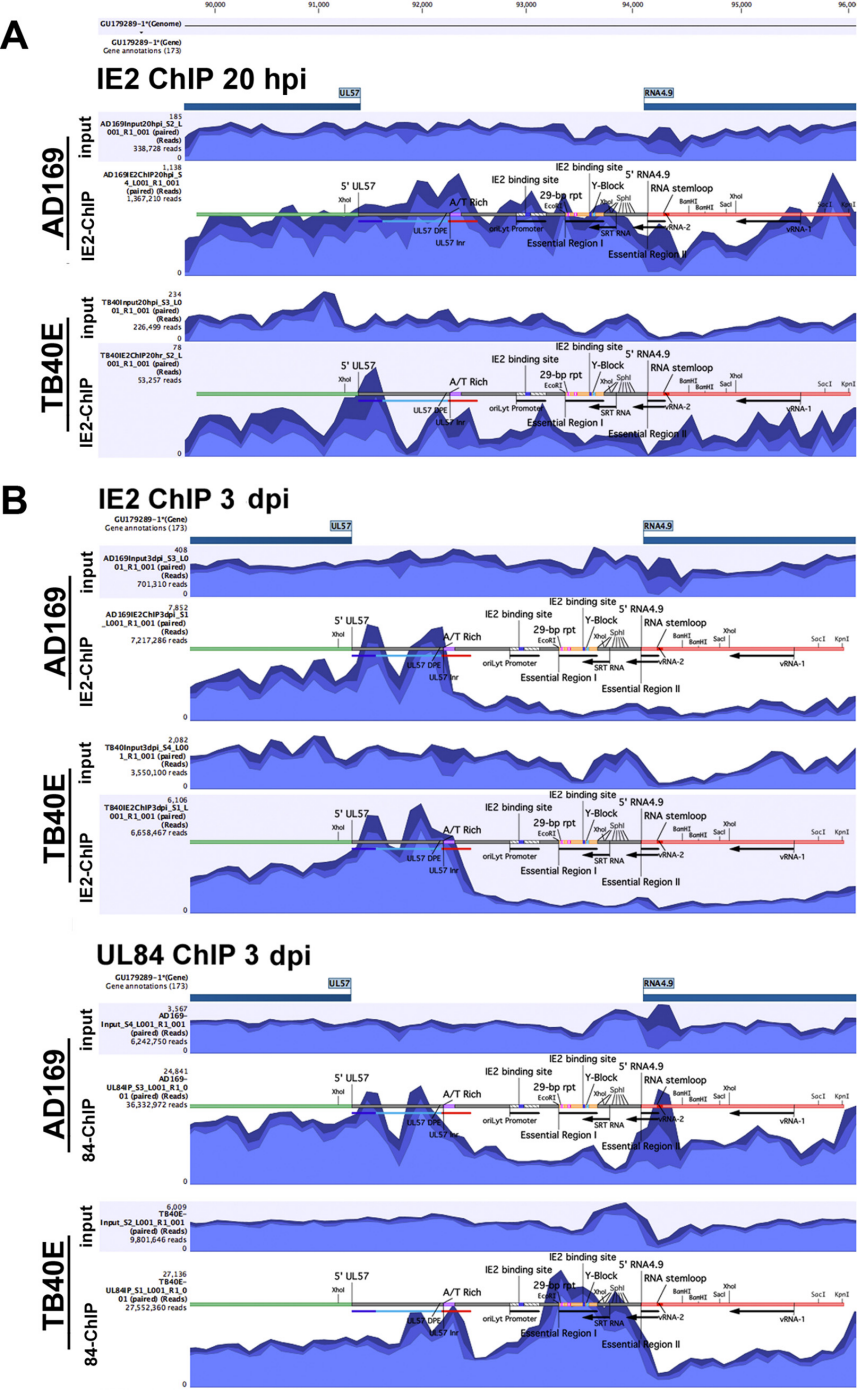
Mapping of IE2 and UL84 ChIP-seq to *ori*Lyt in AD169 and TB40E. The enrichment of UL84 or IE2 to *ori*Lyt in AD169 and TB40E was determined by ChIP-seq analysis. Reads from input and ChIP libraries were aligned to the viral genome and visualized using CLC genomics workbench. Sequencing aligned to *ori*Lyt, encompassing the region between UL57 and RNA4.9, is depicted for input and ChIP samples at (A) 20 hpi for the IE2 ChIP and (B) 3 dpi for the IE2 and UL84 ChIP. Known elements within this region include UL57 (single-stranded binding protein) downstream promoter element (DPE), UL57 initiator region (Inr), 29-bp repeat (rpt), small replication transcript (SRT), virus-associated RNA (vRNA-1 and vRNA-2), essential region I and II, A/T-rich region, Y-block, IE2-binding sites within the *ori*Lyt promoter, RNA stem-loop of the RNA-DNA hybrid, and SphI restriction enzyme sites.

We assessed differences between the AD169 and TB40E ChIP-seqs and focused on the interactions at MIEP and *ori*Lyt sites (Fig. 13 and 14). At the MIEP, in ChIP for both IE2 and UL84, there were areas of enrichment upstream of UL122/UL123, IE1, and IE2 genomic loci (Fig. 13A and B). There were no discernible differences in peak enrichment between AD169 and TB40E in the IE2 ChIP or the UL84 ChIP at the MIEP.

For *ori*Lyt, a schematic was overlaid on ChIP-seq tracks to define where peak enrichments occurred within previously identified elements (Fig. 14). Abbreviations located on the schematic are as follows: UL57 (single-stranded binding protein) downstream promoter element (DPE), UL57 initiator region (Inr), repeat (rpt), small replication transcript (SRT), and virus-associated RNA (vRNA-1 and vRNA-2). Additionally, essential regions I and II, A/T-rich region, the Y-block, IE2-binding sites within the *ori*Lyt promoter, RNA stem-loop of the RNA-DNA hybrid, and restriction enzyme sites are indicated. As previously noted, at 20 hpi, AD169 IE2 reads had a mapped percentage higher than that of TB40E, which may partially explain the slight differences between the IE2-ChIP in AD169 and that in TB40E at early time points. At 20 hpi, AD169 showed a broad area of enrichment from UL57 at the right side of *ori*Lyt to the SphI cluster at the left side, with areas of higher peaks in between. It is also interesting to note the peak at vRNA-1. For TB40E at 20 hpi, there was a similar trend, with notable peaks within the UL57 5′ region, DPE and Inr, and *ori*Lyt promoter (Fig. 14A). By 3 dpi, the IE2-ChIP were similar in AD169 and TB40E, with the predominant areas of enrichment for IE2 between UL57 and A/T-rich region, within the UL57 promoter (Fig. 14B).

Surprisingly, the peak enrichment from the UL84-ChIP revealed some differences of binding between strains. While both AD169 and TB40E showed peak areas of enrichment between the UL57 promoter and the A/T-rich region, the AD169 UL84-ChIP showed a narrow peak at the RNA stem-loop, and TB40E UL84-ChIP did not have a peak at the RNA stem-loop but did have a broader enrichment peak encompassing essential region I and SRT RNA region (Fig. 14B). Essential region I consists of DNA repeat elements, a G/C rich region (Y-block), and an IE2 binding site (11).

### TB40E Δ84 virus can direct DNA synthesis using AD169 *ori*Lyt.

**FIG 15:**
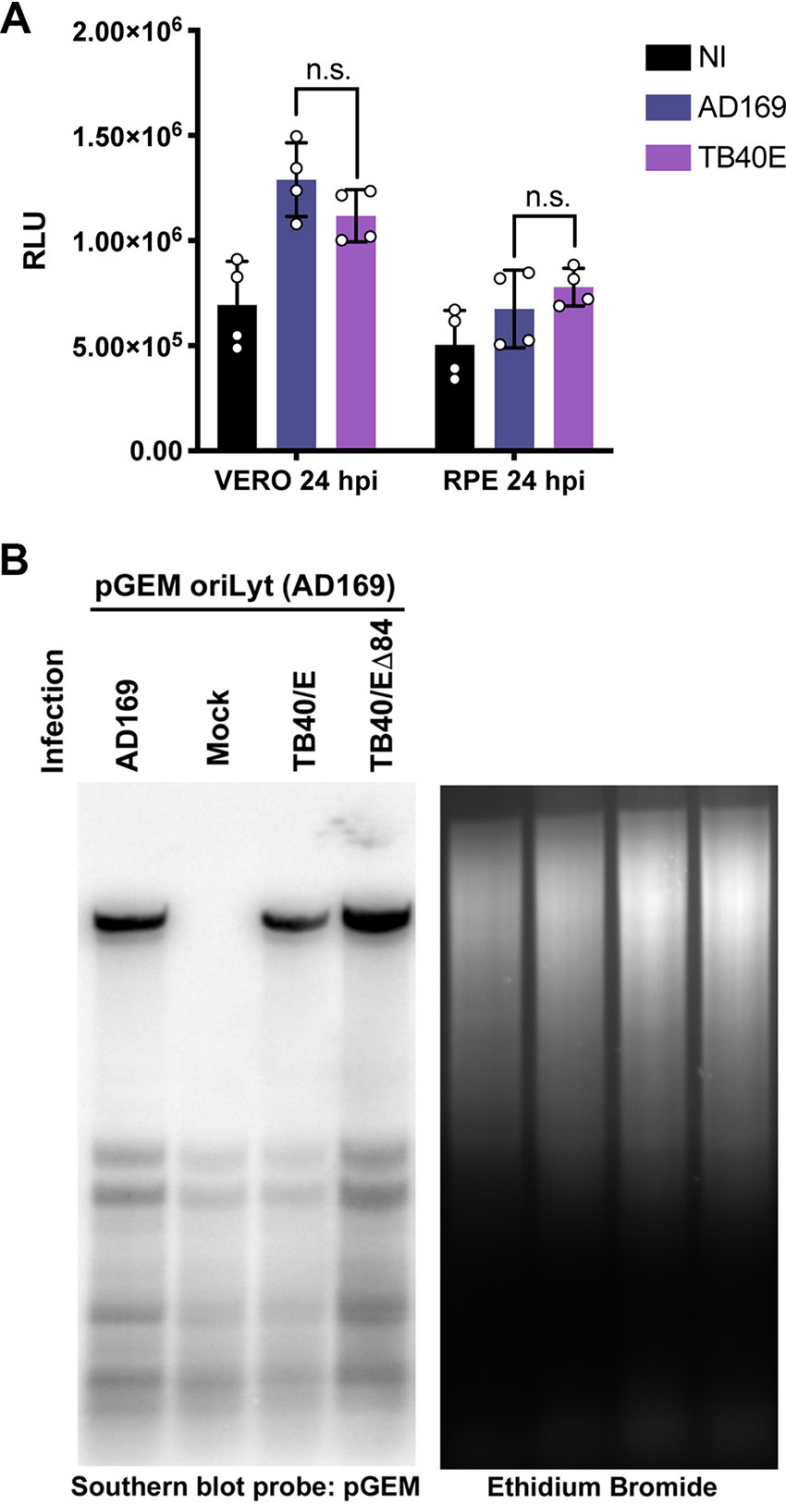
TB40E Δ84 can direct *ori*Lyt-dependent DNA synthesis using *ori*Lyt from AD169. (A) Promoter activity of *ori*Lyt during an infection with AD169 and TB40E. RPE and Vero cells were transfected with luciferase reporter plasmid pGL2-*ori*Lyt. Twenty-four hours posttransfection, cells were infected with either AD169 or TB40E or were noninfected (NI). Cells were harvested 24 hpi and luciferase assay was performed. Error bars indicate SD from independent experiments (*n* = 4). Statistical analysis was performed using one-way ANOVA; n.s., not significant. (B) A transient cotransfection DpnI sensitivity replication assay was performed in HF cells transfected with 20 μg of pGEM-*ori*Lyt (containing *ori*Lyt from AD169) followed by infection with AD169 WT, TB40E WT, TB40E Δ84 (MOI = 10), or mock. Total DNA was harvested and cleaved by restriction enzyme digest with DpnI and EcoRI, followed by DNA separation via agarose gel electrophoresis and transfer to a nylon membrane. Southern blotting was performed by hybridizing a radioactive probe complementary to pGEM DNA and imaged using a phosphorimager.

Since the ChIP-seq analysis revealed differences in UL84 binding to *ori*Lyt, *ori*Lyt activity between UL84-independent and UL84-dependent strains was further tested in the context of infection. A luciferase assay was used to compare *ori*Lyt activity between AD169 and TB40E in both RPE and Vero cells during an infection (Fig. 15A). For this assay, the pGL2-*ori*Lyt luciferase plasmid was transfected into RPE and Vero cells 24 h after transfection cells were infected with either AD169 or TB40E (MOI = 4), and luciferase assay was performed 24 hpi. There were no significant differences in activity of the *ori*Lyt promoter in cells infected with TB40E compared to that in cells infected with AD169.

To determine if the *ori*Lyt sequence itself contains *cis*-elements that contribute to the differences in the requirement for IE2 and UL84 to act as initiator proteins for viral DNA synthesis, a transient cotransfection replication assay was performed (9, 19) (Fig. 15B). In the transient cotransfection replication assay, a plasmid (pGEM-*ori*Lyt) containing the *cis*-acting *ori*Lyt from AD169 was transfected into HF cells, followed by infection with AD169, TB40E, or TB40E Δ84. Following infection, total DNA was harvested and subjected to restriction digest with restriction endonuclease designation (EcoRI) and DpnI. DNA was separated on an agarose gel and transferred to a membrane before hybridization with a ^32^P-labeled pGEM DNA probe. Replicated pGEM-*ori*Lyt is resistant to digestion by DpnI and will migrate slower than the transfected DNA which is sensitive to DpnI digestion. There was no replicated pGEM-*oriL*yt detected in mock-infected cells. This demonstrated that cellular factors alone cannot initiate *ori*Lyt-dependent DNA replication. In both AD169 and TB40E infections, amplification of pGEM-*ori*Lyt was detected. Interestingly, pGEM-*ori*Lyt was also amplified in the presence of TB40E Δ84 infection. This indicates that there is not a *cis*-sequence located within AD169 *ori*Lyt that requires UL84 for replication.

## DISCUSSION

For all herpesviruses, origin-dependent DNA synthesis requires a complex of replication proteins. This complex consists of the replication fork machinery that directly participates in DNA synthesis. In addition to these core proteins, an OBP is required to initiate DNA synthesis by recognizing the viral DNA origin to facilitate recruitment of the replication factors to that site. Initiation of lytic DNA replication at the viral origin is a highly regulated event. For HCMV, there have been conflicting reports on the ability of UL84 and IE2 to act as the OBP to initiate origin-dependent DNA replication. Many of those reported differences are due to varying cell type and also whether the proteins are expressed from native promoters or constitutive promoters (9, 11, 17, 40). In order to define the minimal *trans*-acting factors that directly participate in *ori*Lyt-dependent DNA replication, viral factors were expressed from a constitutive promoter to determine which proteins were dispensable and acting purely as transactivators for the native promoter. Sarisky and Hayward used HCMV core replication proteins expressed from constitutive promoters and showed that in HF cells, UL84, IE2, and UL36-38 were required (17). In Vero cells, they determined that UL84 was the only noncore protein required for initiation of viral DNA synthesis, although activity was increased by the presence of IE2, UL112-113, and UL69 (17). They also demonstrated that when using the Epstein-Barr virus (EBV) core replication machinery, UL84 was sufficient to initiate *ori*Lyt-dependent DNA replication. Subsequently, using the core replication machinery from herpes simplex virus 1 (HSV-1) demonstrated that omission of UL84 from the cotransfection replication assay in Vero cells had no effect upon *ori*Lyt-dependent DNA synthesis and concluded that the IE2-86 was most likely the initiator protein (40).

It is interesting to note that the set of core replication proteins are functionally interchangeable between different human herpesviruses in transient assays but the initiator OBP is unique and will recognize only the origin from its specific virus. Using this approach has allowed the OBP to successfully be identified in some herpesviruses. For EBV DNA replication, the core proteins and three additional factors, Zta (BZFL1), Rta, and Mta, were originally identified (41, 42). Using proteins expressed from constitutive promoters along with the HSV-1 core replication factors, the only protein that proved to be indispensable was Zta as the OBP (42). This same approach was attempted to identify the HCMV OBP. First, using the EBV core proteins in Vero cells, UL84 was suggested as the required OBP (17). Later studies using the HSV-1 core proteins in HF cells suggested IE2 as the required OBP (40).

Early studies hypothesized that UL84 was the OBP for HCMV and functioned similarly to HSV-1 OBP, UL9. For HSV-1, UL9 is the only additional viral protein, along with the six core replication proteins, that is required to replicate the HSV lytic origin (43, 44). UL9 has a variety of functions, including origin-specific DNA-binding, ATPase, and helicase activities (45–47). UL9 also interacts with UL42 (DNA polymerase processivity factor), ICP8 (UL29 single-stranded DNA [ssDNA] binding protein), and UL8 (component of the helicase/primase complex). It is thought that the interaction between UL42 and UL9 helps to load the viral processivity factor onto DNA (48–50). The interaction between ICP8 and UL9 has been reported to stimulate the helicase and ATPase activities of UL9 (51). As HSV is the prototypic herpesvirus, it was assumed that HCMV would be similar and encode a single OBP. Both UL84 and IE2 have been reported to interact with UL44 (DNA polymerase processivity factor) (31, 36–38). The interaction of UL84 and IE2 with additional HCMV core replication proteins has not been described extensively.

In many models of origin-dependent DNA replication, the act of transcription is often linked with initiation of DNA replication as a way to help unwind specific regions of DNA and facilitate the assembly of the core replication complex. The ability of the OBP to facilitate transcription within the origin has been shown to be required for both HCMV and EBV, with experiments inserting a strong constitutive promoter within *ori*Lyt and alleviating the need for additional viral transactivators, Zta for EBV and IE2 for HCMV (16, 52). The ability of IE2 to transactivate viral promoters may partly explain its role in origin-dependent DNA replication. Specific IE2 binding sites have been found in promoters activated by IE2 (53–56).

In striking contrast to its role as a transactivator, IE2 also has the ability to repress specific promoters, namely, the MIE promoter and a promoter within *ori*Lyt. Within the MIE promoter, IE2 binds to the *cis*-repressive sequence (crs) located between −13 and +1 upstream of the initiator site and negatively autoregulates the MIEP activity (54, 57–59). There is also a bidirectional promoter located within *ori*Lyt, which is repressed by IE2 (16). In transient assays using luciferase expression to measure the relative promoter activity, both the MIE and *ori*Lyt promoters are constitutively active and repressed by IE2, and the addition of UL84 relieves the IE2-mediated repression (16). It was reported that the ability of IE2 does not directly bind to the *ori*Lyt promoter but may be dependent on an unknown viral or cellular factor (16). IE2 protein interacts with a number of different viral proteins, including itself and UL84 (31, 32, 60, 61). Dominant negative inhibitors that block the ability of IE2 and UL84 to heterodimerize and UL84 to oligomerize have been used to disrupt viral DNA synthesis, strongly suggesting that those interactions play an important role in viral replication (34). The interaction between UL84 and IE2 causes a decrease in the ability of IE2 to transactivate early viral promoters, and the overexpression of UL84 before infection results in a decrease in viral replication (33).

It was noted previously that mutations within UL84 ORF accumulate in TR and TB40E-BAC strains when grown in RPE cells (62). The mutations resulted in loss of C-terminal residues, which are involved in IE2-mediated repressive activity of UL84. It was hypothesized that these may result in a growth advantage *in vitro*. The ability for TB40E IE2 (H388D) to interact with promoters in a manner different from that of other strains of HCMV was first alluded to by Spector and Yetming (63). They noted an increase in the accumulation of IE1 and IE2 proteins in the TB40E ΔUL84 virus compared to that in the wild type. Since IE2 is known to negatively autoregulate the MIE promoter and decrease the expression of IE2, the observation that TB40E ΔUL84 had an increase in protein of IE1 and IE2 suggested that there was increased activity (or decrease in repression) of the MIE promoter. The function of IE2 in initiation of *ori*Lyt-dependent DNA replication has taken on new significance in light of the ability of TB40E to replicate without UL84.

The exact mechanism of initiation of lytic DNA synthesis in HCMV continues to remain an enigma. However, the data presented here suggest that there may be differences in the ability of TB40E IE2 to repress the *ori*Lyt promoter and UL84 to relieve the IE2 mediated repression. The luciferase assay of the *ori*Lyt promoter with IE2 alone in T98G and Vero cells suggested that IE2 from TB40E had a significant difference and allowed for more transcriptional activity of the promoter compared to AD169. However, in the presence of UL84, there was no difference in these cell types, but there was a significant difference in *ori*Lyt activity in RPE, with higher activity in the presence of AD169 IE2 and UL84 compared to that in the presence of TB40E IE2 and UL84. With these discordant data, we acknowledge the limitations in interpreting the results of the transfection luciferase systems. This led us to suspect potential differences in *cis*-elements within *ori*Lyt that may be differentially recognized by either UL84 or IE2 from AD169 and TB40E. To explore this possibility, we turned to the DpnI sensitivity replication assay using a plasmid containing AD169 *ori*Lyt and infection with TB40E ΔUL84. If AD169 *ori*Lyt did contain a *cis*-element that required UL84 in order to facilitate initiation of DNA synthesis, we would have expected TB40E ΔUL84 to be unable to direct *ori*Lyt-dependent DNA synthesis. Interestingly, we found that TB40E ΔUL84 could replicate AD169 *ori*Lyt, which suggests that either TB40E IE2 can functionally replace UL84 during *ori*Lyt-dependent initiation or TB40E UL84 plays a different role during DNA synthesis where the interaction with *ori*Lyt is not required. Following the potential differences noted in the ChIP-seq at *ori*Lyt, additional studies will be needed to determine quantitative differences or variations in affinity of IE2 and UL84 for *ori*Lyt DNA between AD169 and TB40E. The larger question of the clinical consequences of UL84-independent strains remains unknown but is an area to be further explored with relevant models of infection and pathogenesis.

## MATERIALS AND METHODS

### Cells and viruses.

HF, RPE, 293FT, Vero, T98G, T-HF, and T-HF UL84 cells were cultured in Dulbecco’s modified Eagle medium (DMEM) supplemented with 10% fetal bovine serum (Corning). Cells were maintained at 37°C in a 5% CO_2_ environment. The AD169 WT, AD169 ΔUL84, TB40E WT, TB40E ΔUL84, and TB40E ΔUL84 IE2 D388H viral mutants were kept as frozen stocks at −80°C. Virus stocks were grown and titrated on HF or T-HF 84 cells for virus mutants that required complementation with UL84.

### UL84-expressing T-HF cell line.

To produce the constitutive UL84-expressing T-HF cell line, the UL84 gene was cloned into the pLVX-EF1α-IRES-Puro lentivirus vector (Clontech) according to the manufacturer’s instructions. The pLVX vector was linearized by EcoRI restriction enzyme digest followed by gel purification. Primers were designed with homology to the UL84 locus with an in-frame FLAG tag (Table 1). The UL84 PCR product was treated with DpnI to remove template DNA, followed by gel purification. GeneArt seamless cloning kit (Life Technologies) was used to insert the UL84 PCR product into the pLVX linear vector, and the cloning product was transformed in TOP 10 Escherichia coli (Thermo Fisher). After incubating the transformed E. coli overnight on LB-agar carbenicillin (50 μg/ml), colonies were picked and screened by restriction enzyme digestion for correct insertion of the UL84 gene into pLVX vector. From the correct colonies, a cesium chloride (CsCl) maxiprep was performed to isolated pLVX-EF1α UL84 plasmid DNA to be used for transfection. The transfection was performed with the lentivirus packaging mix supplied by the manufacturer along with their transfection reagent. After transfection, the HEK293 cells were incubated for 72 h before the lentivirus was isolated and concentrated using the Lenti-X concentrator (Clontech) according to the manufacturer’s instructions. The purified pLVX-EF1α UL84 lentivirus was used to transduce T-HF cells. Forty-eight hours after transduction, the T-HF cells were selected with 0.5 μg/ml of puromycin (Invivogen). The puromycin-containing medium was refreshed every 3 days, and colonies of puromycin-resistant cells were allowed to grow out for 10 to 12 days before being trypsinized and replated into new tissue culture flasks. Once the cells had grown confluent, UL84 expression was screened by Western blotting.

**TABLE 1:** Primer and gBlock sequences

Name	Type	Sequence^*a*^
pLVX-EF1α UL84	FWD	5′-TTCCATTTCAGGTGTCGTGAGGATCTATTTCCGGTTCGAATTCTATGCCACGCGTCGACCCCAACCTTCG-3′
pLVX-EF1α UL84	REV	5′-CGGGATCCGCGGCCGCTCTAGAACTAGTCTCGAGGGAATTCCTTACTTATCGTCGTCATCCTTGTAATCGAGATCGCCGCAGACCATGGC-3′
pGL2-MIEP	FWD	5′-TGAGCTAACATAACCCGGGAGGTACCTCAGATCGCCTGGAGACGCC-3′
pGL2-MIEP	REV	5′-AAGCTTACTTAGATCTCGAGCTAGCAGGCAGAGGACTCCATCGTGT-3′
pGL2-*ori*Lyt	gBlock	5′-TGAGCTAACATAACCCGGGAGGTACCCGCGGTAGAATACAGCGATCCCTAGTGAAGCCACACCCATTACGTGTAGCCATATCCGCTTACGTATACAGCCACACCCCTAGGTACGCCACCTTATCTACCAATCACAGAAACGGATATACAATGACCCCTCCCTAGACTCCACCCCTTGTACGGAAATTTCAGATAGGTGGAACCCGTTAGGGTTCCACCGTCCTCGGTGTACGTACAGGCTTCTCCGTCTACCGGAAATATACACCTGCTGACGTAGACGCTACTCCCGGATACGCGTCATAAGCTTGCTAGCTCGAGATCTAAGTAAGCTT-3′
pSi-UL84 (TB40E)	FWD	5′-TATAGGCTAGCCTCGAGAATTCATGCCACGCGTCGACCCCAAC-3′
pSI-UL84 (TB40E)	REV	5′-GAAGCGGCCGCCCGGGTCGACTTTAGAGATCGCCGCAGACCAT-3′
pSI-IE2 (TB40E)	gBlock	5′-TATAGGCTAGCCTCGAGAATTCATGGAGTCCTCTGCCAAGAGAAAGATGGACCCTGACAACCCTGACGAGGGCCCTTCCTCCAAGGTGCCACGGCCCGAGACACCCGTGACCAAGGCCACGACGTTCCTGCAGACTATGTTAAGGAAGGAGGTTAACAGTCAGCTGAGCCTGGGAGACCCGCTGTTCCCAGAATTGGCCGAAGAATCTCTCAAAACCTTTGAACAAGTGACCGAGGATTGCAACGAGAACCCCGAAAAAGATGTCCTGGCAGAACTCGGTGACATCCTCGCCCAGGCTGTCAATCATGCCGGTATCGATTCCAGTAGCACCGGCCCCACGCTGACAACCCACTCTTGCAGCGTTAGCAGCGCCCCTCTTAACAAGCCGACCCCCACCAGCGTCGCGGTTACTAACACTCCTCTCCCCGGGGCATCCGCTACTCCCGAGCTCAGCCCGCGTAAGAAACCGCGCAAAACCACGCGTCCTTTCAAGGTGATTATTAAACCGCCCGTGCCTCCCGCGCCTATCATGCTGCCCCTCATCAAACAGGAAGACATCAAGCCCGAGCCCGACTTTACCATCCAGTACCGCAACAAGATTATCGATACCGCCGGCTGTATCGTGATCTCTGATAGCGAGGAAGAACAGGGTGAAGAAGTCGAAACCCGCGGTGCTACCGCGTCTTCCCCTTCCACCGGCAGCGGCACGCCGCGAGTGACCTCTCCCACGCACCCGCTCTCCCAGATGAACCACCCTCCTCTTCCCGATCCCTTGGGCCGGCCCGATGAAGATAGTTCCTCTTCGTCTTCCTCCTCCTGCAGTTCGGCTTCGGACTCGGAGAGTGAGTCCGAGGAGATGAAATGCAGCAGTGGCGGAGGAGCATCCGTGACCTCGAGCCACCATGGGCGCGGCGGTTTTGGTGGCGCGGCCTCCTCCTCTCTGCTGAGCTGCGGCCATCAGAGCAGCGGCGGGGCGAGCACCGGACCCCGCAAGAAGAAGAGCAAACGCATCTCCGAGTTGGACAACGAGAAGGTACGCAATATCATGAAAGATAAGAACACCCCCTTCTGCACACCCAACGTGCAGACTCGGCGGGGTCGCGTCAAGATTGACGAGGTGAGCCGCATGTTCCGCAACACCAATCGCTCTCTTGAGTACAAGAACCTGCCCTTCACGATTCCCAGTATG**GAC**CAGGTGTTAGATGAGGCCATCAAAGCTTGCAAAACCATGCAGGTGAACAACAAGGGCATCCAGATCATCTACACCCGCAATCATGAGGTGAAGAGTGAGGTGGATGCGGTGCGGTGTCGCCTGGGCACCATGTGCAACCTGGCCCTCTCCACTCCCTTCCTCATGGAGCACACCATGCCTGTGACACACCCACCCGAAGTGGCGCAGCGCACGGCCGATGCTTGTAACGAAGGCGTCAAAGCCGCGTGGAGCCTCAAAGAATTGCACACCCACCAATTATGCCCCCGTTCTTCCGATTACCGCAACATGATCATCCACGCTGCCACCCCCGTGGACCTGTTGGGCGCTCTCAACCTGTGCCTACCCCTGATGCAAAAGTTTCCCAAACAGGTCATGGTGCGCATCTTCTCCACCAACCAGGGTGGGTTCATGCTCCTATCTACGAGACGGCCGCGAAGGCCTACGCCGTGGGGCAGTTTGAGCAGCCCACCGAGACCCCTCCCGAAGACCTGGACACCCTGAGCCTGGCCATCGAGGCAGCCATCCAGGACCTGAGGAACAAGTCTCAGTAAAGTCGACCCGGGCGGCCGCTTC-3′
pSI-IE2 (AD169)	gBlock	5′-TATAGGCTAGCCTCGAGAATTCATGGAGTCCTCTGCCAAGAGAAAGATGGACCCTGATAATCCTGACGAGGGCCCTTCCTCCAAGGTGCCACGGCCCGAGACACCCGTGACCAAGGCCACGACGTTCCTGCAGACTATGTTGAGGAAGGAGGTTAACAGTCAGCTGAGTCTGGGAGACCCGCTGTTTCCAGAGTTGGCCGAAGAATCCCTCAAAACTTTTGAACAAGTGACCGAGGATTGCAACGAGAACCCCGAGAAAGATGTCCTGGCAGAACTCGGTGACATCCTCGCCCAGGCTGTCAATCATGCCGGTATCGATTCCAGTAGCACCGGCCCCACGCTGACAACCCACTCTTGCAGCGTTAGCAGCGCCCCTCTTAACAAGCCGACCCCCACCAGCGTCGCGGTTACTAACACTCCTCTCCCCGGGGCATCCGCTACTCCCGAGCTCAGCCCGCGTAAGAAACCGCGCAAAACCACGCGTCCTTTCAAGGTGATTATTAAACCGCCCGTGCCTCCCGCGCCTATCATGCTGCCCCTCATCAAACAGGAAGACATCAAGCCCGAGCCCGACTTTACCATCCAGTACCGCAACAAGATTATCGATACCGCCGGCTGTATCGTGATCTCTGATAGCGAGGAAGAACAGGGTGAAGAAGTCGAAACCCGCGGTGCTACCGCGTCTTCCCCTTCCACCGGCAGCGGCACGCCGCGAGTGACCTCTCCCACGCACCCGCTCTCCCAGATGAACCACCCTCCTCTTCCCGATCCCTTGGGCCGGCCCGATGAAGATAGTTCCTCTTCGTCTTCCTCCTCCTGCAGTTCGGCTTCGGACTCGGAGAGTGAGTCCGAGGAGATGAAATGCAGCAGTGGCGGAGGAGCATCCGTGACCTCGAGCCACCATGGGCGCGGCGGTTTTGGTGGCGCGGCCTCCTCCTCTCTGCTGAGCTGCGGCCATCAGAGCAGCGGCGGGGCGAGCACCGGACCCCGCAAGAAGAAGAGCAAACGCATCTCCGAGTTGGACAACGAGAAGGTGCGCAATATCATGAAAGATAAGAACACCCCCTTCTGCACACCCAACGTGCAGACTCGGCGGGGTCGCGTCAAGATTGACGAGGTGAGCCGCATGTTCCGCAACACCAATCGCTCTCTTGAGTACAAGAACCTGCCCTTCACGATTCCCAGTATG**CAC**CAGGTGTTAGATGAGGCCATCAAAGCCTGCAAAACCATGCAGGTGAACAACAAGGGCATCCAGATTATCTACACCCGCAATCATGAGGTGAAGAGTGAGGTGGATGCGGTGCGGTGTCGCCTGGGCACCATGTGCAACCTGGCCCTCTCCACTCCCTTCCTCATGGAGCACACCATGCCCGTGACACATCCACCCGAAGTGGCGCAGCGCACAGCCGATGCTTGTAACGAAGGCGTCAAGGCCGCGTGGAGCCTCAAAGAATTGCACACCCACCAATTATGCCCCCGTTCCTCCGATTACCGCAACATGATCATCCACGCTGCCACCCCCGTGGACCTGTTGGGCGCTCTCAACCTGTGCCTGCCCCTGATGCAAAAGTTTCCCAAACAGGTCATGGTGCGCATCTTCTCCACCAACCAGGGTGGGTTCATGCTGCCTATCTACGAGACGGCCGCGAAGGCCTACGCCGTGGGGCAGTTTGAGCAGCCCACCGAGACCCCTCCCGAAGACCTGGACACCCTGAGCCTGGCCATCGAGGCAGCCATCCAGGACCTGAGGAACAAGTCTCAGTAAAGTCGACCCGGGCGGCCGCTTC-3′
AD169 Δ84 (for BAC recombineering)	FWD	5′-GCGCGGACGCCTAGTGTCCGTTTCCCATCACCAGGGTCCTCTGTGCTTGGTGTCTGCGGGCGCGAGCGATTTATTCAACAAAGCCACC-3′
AD169 Δ84 (for BAC recombineering)	REV	5′-ATTTAAAGGCTGAGCCGGCCCTCTCGCGCCCGCAGACACCAAGCACAGAGGACCCTGGTGATGGGACGCGTATATCTGGCCCGTACAT-3′
TB40E Δ84 (for BAC recombineering)	gBlock	5′-GCGCGGACGCCTAGTGTCCGTTTTCCATCACCAGGGTCCTCTGTGCTTGGTGTCTGCGGGCGCGAGCGATTTATTCAACAAAGCCACGTTGTGTCTCAAAATCTCTGATGTTACATTGCACAAGATAAAAATATATCATCATGAACAATAAAACTGTCTGCTTACATAAACAGTAATACAAGGGGTGTTATGAGCCATATTCAACGGGAAACGTCTTGCTCGAGGCCGCGATTAAATTCCAACATGGATGCTGATTTATATGGGTATAAATGGGCTCGCGATAATGTCGGGCAATCAGGTGCGACAATCTATCGATTGTATGGGAAGCCCGATGCGCCAGAGTTGTTTCTGAAACATGGCAAAGGTAGCGTTGCCAATGATGTTACAGATGAGATGGTCAGACTAAACTGGCTGACGGAATTTATGCCTCTTCCGACCATCAAGCATTTTATCCGTACTCCTGATGATGCATGGTTACTCACCACTGCGATCCCCGGGAAAACAGCATTCCAGGTATTAGAAGAATATCCTGATTCAGGTGAAAATATTGTTGATGCGCTGGCAGTGTTCCTGCGCCGGTTGCATTCGATTCCTGTTTGTAATTGTCCTTTTAACAGCGATCGCGTATTTCGTCTCGCTCAGGCGCAATCACGAATGAATAACGGTTTGGTTGATGCGAGTGATTTTGATGACGAGCGTAATGGCTGGCCTGTTGAACAAGTCTGGAAAGAAATGCATAAGCTTTTGCCATTCTCACCGGATTCAGTCGTCACTCATGGTGATTTCTCACTTGATAACCTTATTTTTGACGAGGGGAAATTAATAGGTTGTATTGATGTTGGACGAGTCGGAATCGCAGACCGATACCAGGATCTTGCCATCCTATGGAACTGCCTCGGTGAGTTTTCTCCTTCATTACAGAAACGGCTTTTTCAAAAATATGGTATTGATAATCCTGATATGAATAAATTGCAGTTTCATTTGATGCTCGATGAGTTTTTCTAATCAGAATTGGTTAATTGGTTGTAACACTGGCATTACCCTGTTATCCCTAGATCGATGTACGGGCCAGATATACGCGTTCCATCACCAGGGTCCTCTGTGCTTGGTGTCTGCGGGCGCGAGAGGGCCGGCTCAGCCTTTAAAT-3′
TB40E IE2 (D388H) (for BAC recombineering)	gBlock	5′-GCTCTCTTGAGTACAAGAACCTGCCCTTCACGATTCCCAGTATGCACCAGGTGTTAGATGAGGCCATCACGATTTATTCAACAAAGCCACGTTGTGTCTCAAAATCTCTGATGTTACATTGCACAAGATAAAAATATATCATCATGAACAATAAAACTGTCTGCTTACATAAACAGTAATACAAGGGGTGTTATGTCACATATCCAGCGTGAGACGTCATGCTCCCGTCCGCGTCTGAATAGCAACATGGACGCAGATTTGTATGGATACAAGTGGGCGCGTGACAATGTCGGTCAGTCGGGCGCAACTATCTACCGCTTGTACGGGAAACCTGATGCGCCCGAATTGTTCTTGAAGCACGGGAAAGGTTCGGTGGCTAACGATGTAACCGATGAGATGGTACGTTTAAATTGGCTGACGGAGTTCATGCCTTTGCCAACAATTAAGCATTTTATCCGTACTCCTGACGATGCCTGGCTTCTGACCACAGCAATTCCTGGAAAAACCGCTTTCCAAGTCTTAGAGGAGTACCCCGACAGTGGGGAAAATATCGTGGATGCCCTGGCCGTATTTTTGCGCCGCTTACACTCCATCCCTGTGTGTAATTGCCCATTTAACTCGGATCGTGTCTTTCGCTTGGCGCAAGCTCAGTCGCGCATGAACAACGGGTTAGTAGATGCTAGCGATTTTGACGATGAGCGTAATGGCTGGCCGGTCGAGCAGGTGTGGAAGGAGATGCACAAATTATTACCTTTCTCGCCAGACAGTGTAGTCACTCACGGAGATTTCTCCTTGGACAATCTGATCTTCGACGAAGGAAAGTTGATTGGCTGTATCGATGTTGGACGTGTCGGAATTGCTGACCGTTACCAGGATTTAGCAATTCTTTGGAACTGCTTGGGGGAGTTCTCTCCATCGCTGCAAAAGCGTTTGTTCCAAAAATACGGGATCGATAACCCAGATATGAACAAGTTACAGTTCCACTTAATGCTTGACGAGTTCTTTTAATCAGAATTGGTTAATTGGTTGTAACACTGGCATTACCCTGTTATCCCTAGATCGATGTACGGGCCAGATATACGCGGCCCTTCACGATTCCCAGTATGCACCAGGTGTTAGATGAGGCCATCAAAGCTTGCAAAACCATGCAGGT-3′

a The bold and underlined nucleotide sequence is indicating the difference between the IE2 protein from strain TB40B (GAC codes for an aspartic acid D) and AD169 (CAC codes for histidine H).

### Imaging and immunofluorescent assay.

Confluent HF cells were plated onto coverslips and infected with the indicated viral strains (MOI = 4). At 3 dpi, mock and infected HF cells were fixed using 4% paraformaldehyde in phosphate-buffered saline (PBS) for 15 min at room temperature followed by permeabilizing with 0.5% Triton X-100 in PBS for 20 min at room temperature. All washes were performed using 3% bovine serum albumin (BSA) in PBS. For EdU labeling, cells were incubated with 10 μM EdU for 30 min to label nascent viral DNA. Cells were then conjugated to a fluorescent dye by using the Click-It Plus EdU Alexa Fluor 594 imaging kit (catalog number C10693) according to the manufacturer’s protocol. After fixation, permeabilization, and/or EdU labeling, cells were blocked with 3% goat serum for 30 min to 1 h. Target proteins were identified by diluting primary antibody in 3% BSA in PBS. Antibodies used were UL84 1:1,000 (34), UL44 1:500 (Fitzgerald, 10-C50I), IE1 1:1,000 (64), and IE2 1:1,000 (Millipore, MAB8140). Primary antibodies were incubated overnight 4°C, followed by labeling with secondary antibody, Alexa Fluor 594 anti-mouse 1:2,000 (Invitrogen, number A21201). Coverslips were mounted onto glass slides using ProLong Gold Antifade reagent with DAPI (4′,6-diamidino-2-phenylindole; Invitrogen) and imaged at ×63 magnification

### Fast and efficient nascent DNA isolation.

The FENDI protocol was used to isolate EdU-labeled DNA and used for quantitative analysis by qPCR. The protocol was followed as described previously (26).

### Plasmids.

pGL2-MIEP and pGL2-*ori*Lyt plasmids were constructed by digesting pGL2 with restriction endonucleases KpnI and NheI. The MIEP was PCR amplified with primers indicated in Table 1. The *ori*Lyt DNA sequence was synthesized as a gBLock (IDT) (Table 1). Ligations were performed using GeneArt Seamless Cloning and Assembly (catalog no. A13288) followed by transformation in One Shot TOP10 chemically competent E. coli (catalog no. C404010) according to the manufacturer’s protocol.

pSI-IE2 (AD169) and pSI-IE2 (TB40E) were constructed by digesting pSI (Promega) with restriction endonucleases Xba I and Mlu I. The IE2 sequences from both HCMV strains were synthesized as gBlocks (IDT) (Table 1). For TB40E, the codon that resulted in an amino acid switch is emphasized in bold. The pSI-UL84 (AD169) was created as published previously (16). The pSI-UL84 (TB40E) was made by PCR amplification corresponding to TB40E BAC template DNA followed by ligation and cloning as discussed above (Table 1). pGL2-UL112/113 construct was described previously (34, 65).

### Co-immunoprecipitation and immunoblotting.

Plasmid transfections were performed using TransIT LT1 reagent in a 1:2 ratio (DNA/reagent) according to the manufacturer’s guidelines (Mirus, MIR2306). Cells were transfected with 6 μg of recombinant plasmid containing UL84 and IE2 (AD169 or TB40E). Empty pGEM-7Zf(−) plasmid was used to normalize the amount of transfected DNA in each sample. After 48 hours posttransfection (hpt), cells were harvested. Infected (MOI = 1) or mock-infected cells were harvested at 6 dpi. Cells from transfected or infected samples were lysed with cold NP-40 lysis buffer (50 mM Tris [pH 8.0], 150 mM NaCl, 5 mM EDTA, 1% NP-40) or radioimmunoprecipitation assay (RIPA) buffer (50 mM Tris [pH 8.0], 150 mM NaCl, 1% Triton X, 1% NP-40, 0.1% SDS, 0.5% sodium deoxycholate, 2 mM EDTA) including protease inhibitor 1:100 (Sigma, P8340) for 5 to 10 min. Cells were scraped and sheared by sonication. Cell debris was pelleted by centrifugation, and the supernatant was incubated with IE2 or UL84 antibody for 4 h at 4°C. A fraction of supernatant was saved for input. DynaBeads protein G (LT-0224; Invitrogen) were washed with 1× PBS before being added to the lysate-antibody mixture (30 μl/IP). Incubation with beads was performed overnight at 4°C. The beads were washed with PBS and protein was eluted using Laemmli buffer with β-mercaptoethanol and boiled at 95°C for 5 min. The denatured samples from Co-IPs and harvested lysate were resolved by SDS-PAGE and transferred to a nitrocellulose membrane. The membrane was blocked in 5% milk in Tris-buffered saline, 0.05% Tween 20 (1× TBST). Primary antibodies were diluted in blocking buffer and incubated overnight at 4°C: UL84 1:5,000, IE2 1:2,000, or β-actin 1:1,000 (Abcam, ab8224). Protein was detected by incubation with secondary antibody using IRDye 680 anti-mouse Ab 1:10,000 (LI-COR, number 926-68070).

### Luciferase assay.

RPE, Vero, or T98G cells were seeded 18 h prior to transfection into 12-well tissue culture plates, at 60 to 70% confluence. Cotransfections were performed as described previously. Plasmids pSI-IE2 (TB40E), pSI-IE2 (AD169), pSI-UL84 (TB40E), and pSI-UL84 (AD169) were cotransfected with PGL2-MIEP, PGL2-*ori*Lyt, or PGL2-UL112/113 luciferase reporter constructs. pGEM-7Zf(−) was used as an empty vector control and used to equalize the amount of DNA in each transfection. For infection studies, cells were infected 24 h posttransfection with AD169 or TB40E (MOI = 4). Cell lysates were harvested 48 to 72 h posttransfection. RPE, Vero, and T98G cells were treated with reporter lysis buffer (Promega) and scraped. Luciferase assay was performed using the Steady-Glo luciferase assay system as recommended by the manufacturer’s protocol (Promega, catalog no. E2510).

### Generation of AD169 Δ84, TB40E Δ84, and TB40E Δ84 IE2 D388H mutants.

The AD169 WT and TB40E WT BACmid DNA were used to generate mutants as described previously (66). In both strains, the UL84 locus was removed by homologous recombination using a kanamycin cassette as a selectable marker. In AD169 Δ84, primers were designed with 44-nucleotide (nt) homology to UL84 locus and with 22-nt homology to pEP Kan-S plasmid (Table 1). The pEP Kan-S plasmid (1 ng) was amplified with these primers using PrimeStar Max DNA polymerase (TaKaRa) to generate a Kan-IsceI cassette with UL84 homologous arms. The PCR product was treated with 1 to 2 μl of DpnI (New England Biolabs) for 1 h at 37°C to remove the template DNA, followed by an additional DpnI treatment. The DNA cassette was purified by agarose gel electrophoresis and isolated using the NucleoSpin gel and PCR clean-up kit (Clontech) according to the manufacturer’s instructions. For TB40E Δ84 and TB40E ΔUL84 IE2 D388H, gBlocks were designed with flanking regions that had UL84 homology and also contained the Kan-IsceI cassette (Table 1).

Homologous recombination was performed in competent E. coli (GS1783) that harbors either the AD169 WT or TB40E WT BAC DNA. The GS1783 competent cells containing the viral BAC DNA were cultured from a single colony and grown in 2 to 3 ml of LB with 30 μg/ml of chloramphenicol shaking at 32°C. A total of 125 μl of the overnight culture was added to 12.5 ml LB with chloramphenicol, grown at 32°C shaking until optical density at 600 nm (OD_600_) reached 0.5 to 0.6, and then immediately transferred into a 42°C water bath for 15 min to induce the expression of the recombination enzymes. The cultures were quickly cooled in an ice bath, and bacteria were pelleted at 4,000 rpm and 4°C for 10 min. The bacteria pellet was washed with 20 ml of ice-cold water and pelleted using the same conditions. After the second wash, bacteria were resuspended in 1 ml of water and transferred to a chilled 1.5- ml microtube. The bacteria were centrifuged at 13,000 rpm for 20 to 30 s at 4°C, and the washes were repeated with ice-cold water two more times. After the last wash, the pellet was resuspended in 100 μl of water for immediate use. For long-term storage, bacteria pellet was resuspended in 10% glycerol frozen in an ethanol dry-ice bath and finally stored at −80°C.

For competent cells that were used immediately, 30 μl of resuspended cells were transferred to a prechilled 1.5-ml microtube with 1 μl of gBlock of PCR product (10 to 30 ng DNA). Competent cells with the purified PCR product were electroporated using the Cell-Porator (Gibco BRL) set at 330 μF capacitance and 400 V with a 2-mm cuvette. Cells were recovered in 1 ml of prewarmed LB, shaking for 1.5 h at 32°C. After recovery, the cells were centrifuged at 4,000 rpm for 4 min. The supernatant was removed and the cells were resuspended in the residual volume (∼50 μl) and plated onto LB-agar plates with chloramphenicol (30 μg/ml) and kanamycin (50 μg/ml) and grown overnight at 32°C. Colonies were picked and grown in LB with chloramphenicol and kanamycin for further analysis. Ten to twelve milliliters of overnight culture were pelleted, and DNA was isolated using alkaline lysis of bacteria and phenol-chloroform extraction to purify the DNA. The DNA was subjected to restriction enzyme digestion and separated on an agarose gel followed by radioactive Southern blotting. After correct deletions of UL84 were identified, the Kan cassette was removed.

To remove the Kan cassette, the IsceI enzyme was induced in the presence of arabinose. First, the bacteria containing the mutant BAC DNA were grown as described using 0.5 ml of overnight culture diluted in 10 ml LB with chloramphenicol until an OD_600_ of 0.5 to 0.6 was reached. Two milliliters of culture was diluted in 2 ml of LB with 2% l-arabinose (Sigma-Aldrich) to create a final concentration of 1% arabinose. The diluted cultures were grown at 32°C for 1 h and then incubated in a 42°C water bath for 20 min to induce the expression of the recombination enzymes. The cultures were cooled and returned to the 32°C shaker incubator for an additional 3 to 3.5 h. A total of 100 μl of a 1:1,000 dilution of the cultures was spread onto LB-agar plates containing 1% arabinose and chloramphenicol (30 μg/ml). Plates were incubated overnight at 32°C. Colonies were picked and grown in LB with chloramphenicol for further analysis as described above. BAC DNA was purified using NucleoBond Xtra BAC kit (Clontech) according to the manufacturer’s protocol.

To produce virus, BAC-derived viral DNA was transfected into HF cells using the human dermal fibroblast nucleofector kit (Lonza) along with 0.5 μg pp65 expression plasmid. Recombinant Δ84 UL84-dependent viral strains were propagated on a UL84-expressing cell line (T-HF 84), and UL84-independent strains were propagated on HF cells. After 12 to 14 days of infection, cells were scraped and subjected to three freeze-thaw cycles to release virus from the cells. Cell debris was centrifuged at 4,000 rpm for 10 min twice. Virus was then pelleted at 25,000 rpm for 2 h at 10°C. Viral titer was determined with standard plaque assay on HF cells.

BAC mutants were sequenced at the Nevada Genomics Center on an Illumina NextSeq. FASTQ files were aligned to AD169 and TB40E reference genomes using Qiagen CLC genomics workbench. Raw sequencing data from the viral mutants discussed in this study have been deposited in NCBI’s Sequence Read Archive (SRA) and are accessible with the following accession numbers: AD169 Δ84 (SRR13285300), TB40E Δ84 (SRR13285296), TB40E IE2 D388H (SRR13285298), and TB40E Δ84 IE2 D388H (SRR13285299).

### UL84 and IE2 chromatin-immunoprecipitation sequencing.

Confluent HF cells were infected with AD169 WT, TB40E WT, and TB40E ΔUL84 (MOI = 4). After 20 hpi or 3 dpi, ChIP was performed using Diagenode iDeal ChiP-seq kit for transcription factors according to the manufacturer’s protocol (Diagenode, catalog no. C01010055). Immunoprecipitation reaction mixtures were incubated overnight with 5 μl of UL84 antibody, IE2 antibody, or IgG control antibody. Purified DNA was quantified using the Qubit dsDNA high-sensitivity assay (Thermo Fisher). Libraries were created from ChIP DNA and input DNA samples using the NEXTflex Illumina ChIP-seq library preparation kit (Bioo Scientific, catalog no. 5143-01). Libraries were sequenced with Illumina NextSeq 500/550 mid output kit v2 at the Nevada Genomics Center. ChIP-seq data analysis was performed using Qiagen CLC genomics workbench. Parameters for analysis include the following: reference GU179289.1, no masking, mismatch cost of 3, cost of insertions and deletions equal to linear gap cost, insertion/deletion cost of 3, insertion/deletion open cost of 6, insertion/deletion extend cost of 1, length fraction of 0.8, similarity fraction of 0.9, and nonspecific match handling set to map randomly. Raw data from the ChIP analysis have been deposited in NCBI’s Gene Expression Omnibus (GEO) and are accessible with the following GEO series accession number (GSE): GSE169634.

### DpnI sensitivity replication assay.

Calcium phosphate transfection was performed using approximately 0.5 × 10^6^ HF cells with 20 μg of pGEM-oriLyt DNA. Twenty hours after transfection, cells were washed 5 times with DMEM to remove precipitate, followed by infection with AD169 WT, TB40E WT, or TB40E Δ84 (MOI = 10). At 5 dpi, total DNA was harvested using DNA lysis buffer (2% SDS, 10 mM EDTA, 10 mM Tris-HCl [pH 7.4]) with the addition of 25 μg proteinase K and incubated for 2 h at 65°C, followed by phenol-chloroform extraction and ethanol precipitation. The DNA pellet was resuspended in 1× Tris-EDTA (TE; pH 7.4) and digested with EcoRI and DpnI for 1 h in 37°C. DNA was separated on 0.8% agarose gel in 1× Tris-acetate-EDTA (TAE) buffer and visualized by ethidium bromide staining. The gel was denatured (0.25 M HCl, 15 min) and transferred to Zeta-Probe GT blotting membrane (Bio-Rad) using alkaline capillary transfer (0.4 M NaOH, overnight). pGEM DNA probes were radiolabeled with [^32^P]dCTP (Perkin Elmer) and Rediprime II random prime labeling system (GE Healthcare). Hybridization of the probe to the membrane was performed rotating overnight at 65°C in hybridization buffer (10% polyethylene glycol, 7% sodium dodecyl sulfate [SDS], 1.5× SSPE [1× SSPE is 0.18 M NaCl, 10 mM NaH_2_PO_4_, and 1 mM EDTA {pH 7.7}]). The blots were washed twice for 15 min with 2× SSC-0.1% SDS (1× SSC is 0.15 M NaCl plus 0.015 M sodium citrate) and twice for 30 min with 0.1× SSC-0.1% SDS at 65°C. The blots were imaged using a phosphoimager (GE Healthcare).

### Data availability.

Raw data from the ChIP analysis have been deposited in NCBI’s Gene Expression Omnibus (GEO) and are accessible with the following GEO series accession number (GSE): GSE169634.
